# Metagenome from a *Spirulina* digesting biogas reactor: analysis via binning of contigs and classification of short reads

**DOI:** 10.1186/s12866-015-0615-1

**Published:** 2015-12-17

**Authors:** Vimac Nolla-Ardèvol, Miriam Peces, Marc Strous, Halina E. Tegetmeyer

**Affiliations:** Institute for Genome Research and Systems Biology, Center for Biotechnology, Office G2-152, Bielefeld University, Universitätsstraße 27, Bielefeld, D-33615 Germany; Department of Chemical Engineering, University of Barcelona, C/ Martí i Franquès, 1, 6th floor, Barcelona, 08028 Spain; Centre for Solid Waste Bioprocessing, Schools of Civil and Chemical Engineering, University of Queensland, Brisbane, 4072 QLD Australia; Department of Geoscience, University of Calgary, 2500 University Drive NW, T2N 1 N4, Calgary, AB Canada; Max Planck Institute for Marine Microbiology, Celsiusstraße 1, Bremen, D-28359 Germany

**Keywords:** Metagenome, Metagenome comparison, *Spirulina*, Biogas, Binning, Microbial community

## Abstract

**Background:**

Anaerobic digestion is a biological process in which a consortium of microorganisms transforms a complex substrate into methane and carbon dioxide. A good understanding of the interactions between the populations that form this consortium can contribute to a successful anaerobic digestion of the substrate.

In this study we combine the analysis of the biogas production in a laboratory anaerobic digester fed with the microalgae *Spirulina*, a protein rich substrate, with the analysis of the metagenome of the consortium responsible for digestion, obtained by high-throughput DNA sequencing. The obtained metagenome was also compared with a metagenome from a full scale biogas plant fed with cellulose rich material.

**Results:**

The optimal organic loading rate for the anaerobic digestion of *Spirulina* was determined to be 4.0 g *Spirulina* L^−1^ day^−1^ with a specific biogas production of 350 mL biogas g *Spirulina*^*−1*^ with a methane content of 68 %.

Firmicutes dominated the microbial consortium at 38 % abundance followed by Bacteroidetes, Chloroflexi and Thermotogae. Euryarchaeota represented 3.5 % of the total abundance. The most abundant organism (14.9 %) was related to *Tissierella*, a bacterium known to use proteinaceous substrates for growth. Methanomicrobiales and Methanosarcinales dominated the archaeal community. Compared to the full scale cellulose-fed digesters, Pfam domains related to protein degradation were more frequently detected and Pfam domains related to cellulose degradation were less frequent in our sample.

**Conclusions:**

The results presented in this study suggest that *Spirulina* is a suitable substrate for the production of biogas. The proteinaceous substrate appeared to have a selective impact on the bacterial community that performed anaerobic digestion. A direct influence of the substrate on the selection of specific methanogenic populations was not observed.

**Electronic supplementary material:**

The online version of this article (doi:10.1186/s12866-015-0615-1) contains supplementary material, which is available to authorized users.

## Background

The problems associated with climate change, and the limited supply of fossil fuels has led to an increasing interest in renewable energy sources. One of these alternative energy sources is biogas (a mixture of mainly methane and carbon dioxide) which is obtained through the anaerobic digestion of organic matter [1]. In recent years, energy crops, crops used to produce energy in form of biofuels, have contributed over 50 % of the total biogas production [2]. However, the use of such crops as substrate for biogas production has several drawbacks: (i) use of arable land; (ii) consumption of large quantities of water and (iii) increased use of fertilizers [3, 4]. An alternative to energy crops could be the use of algal biomass. This would overcome the main problems mentioned above; algae do not compete for arable land and with algae it is possible to close the water and nutrient balances [4].

Anaerobic digestion of the microalga *Spirulina* was studied in the late 80s by several authors [5–7]. However, the circumstances at that time, low oil prices and less environmental concerns, led to a loss of interest. The need to use non-fossil energy sources and the biorefinery concept has brought back the attention to using algal biomass to produce biofuels [4, 8–10]. In this context, the use of the microalga *Spirulina* as substrate for the production of biogas has again become an interesting option.

Anaerobic digestion is a biological process in which a wide range of anaerobic bacteria hydrolyze and ferment complex organic compounds first into organic acids, then further to acetate, hydrogen and carbon dioxide, which are subsequently transformed into methane by methanogens [11]. A good understanding of the community structure and the functional interactions between the involved microbial populations, can contribute to the optimization of the anaerobic digestion of the desired substrate. High-throughput DNA sequencing technologies and their application for metagenome analysis have greatly enhanced the study of microbial communities of environmental samples. Several metagenome studies both of biogas producing plants and lab scale anaerobic digesters have been performed to date [12–16]. Moreover, a recent work by Wirth et al*.,* 2015 [17] studied changes in the metagenome of a mesophilic biogas reactor fed with *Scenedesmus obliquus* green algae.

In the present study we combine the analysis of the anaerobic digestion process of *Spirulina* with the analysis of the metagenome from the microbial community in a laboratory digester. Total DNA was extracted from a lab scale bioreactor that converted *Spirulina* into biogas and sequenced using the Ion Torrent (PGM) platform. Sequencing reads were assembled into contigs and these were analyzed with regard to the predicted genes, and by binning to acquire provisional whole genome sequences of abundant community members [18].

In contrast to the cellulose rich substrates commonly used to date in many large scale biogas production plants, *Spirulina* is a protein rich substrate [19]. To determine if the microbial community in the *Spirulina* fed lab-scale digester displays significant adaptation to the substrate, the MG-Rast metagenome analyzer [20] was used to compare the gene content of the obtained metagenome to that of a publicly available metagenome from a fully operative biogas plant fed mainly with cellulose rich material [14].

## Results and discussion

### Biogas production via the anaerobic digestion of *Spirulina*

The anaerobic digestion of freeze dried *Spirulina* was studied using a 2.0 L semi continuous stirred tank reactor (S-CSTR) operated at pH 7.5–8.2, at 37 °C and with a 20-day hydraulic retention time (HRT). After a 71-day start-up period constant daily biogas production (742 ml biogas day^−1^), and constant process parameters (alkalinity, total solids (TS), volatile solids (VS)) were observed, indicating that the bioreactor had reached a pseudo steady state condition. Starting from this pseudo steady state, five different organic loading rates (OLR), from 1.0 to 5.0 g *Spirulina* L^−1^ day^−1^ (dry weight) were studied to determine the optimal OLR for freeze dried *Spirulina*. The biogas production during each period was constant and, as expected, increased when the OLR was increased (Table 1). The biogas production ranged from 470 mL of biogas day^−1^ (69 % of methane) in period I, with an OLR of 1.0 g *Spirulina* L^−1^ day^−1^, up to 2210 mL biogas day^−1^ (62 % methane) in period V, with an OLR of 5.0 g *Spirulina* L^−1^ day^−1^.

**Table 1:** Biogas production and sludge characteristics

Units		Period I	Period II	Period III	Period IV	Period V
	Days	116	74	100	21	29
OLR	g *Spirulina* (L_R_ day)^−1^	1.0	2.0	3.0	4.0	5.0
Daily biogas production	mL biogas day^−1^	470 ± 69	986 ± 176	1487 ± 252	2096 ± 118	2210 ± 325
Daily methane production	mL CH_4_ day^−1^	327 ± 49	648 ± 117	972 ± 190	1397 ± 79	1399 ± 260
CH_4_	%	69 ± 5	69 ± 2	70 ± 13	68 ± 1	62 ± 4
_CO2_	%	30 ± 5	30 ± 2	25 ± 10	31 ± 1	37 ± 4
N_2_;O_2_	%	1 ± 1	1 ± 1	5 ± 16	1 ± 1	1 ± 1
H_2_S	%	<1.0	<1.0	<1.0	<1.0	<1.0
Specific biogas and methane productions						
SBP-VS added	mL_biogas_ (day g VS)^−1^	354 ± 52	369 ± 66	372 ± 63	393 ± 22	334 ± 47
SBP-g *Spirulina* added	mL_biogas_ (day g *Spirulina*)^−1^	313 ± 46	329 ± 58	330 ± 55	349 ± 19	297 ± 42
SMP-VS added	mL_methane_ (day g VS)^−1^	246 ± 37	243 ± 44	243 ± 47	262 ± 14	211 ± 39
SMP-g *Spirulina* added	mL_methane_ (day g *Spirulina*)^−1^	218 ± 33	216 ± 39	216 ± 42	233 ± 13	188 ± 34
Sludge characteristics						
pH		7.5 ± 0.3	7.9 ± 0.1	8.5 ± 0.1	8.6 ± 0.1	8.6 ± 0.1
Alkalinity	g CaCO_3_ L^−1^	8.6 ± 0.7	11.4 ± 0.8	16.5 ± 3.1	20.8 ± 1.2	25.4 ± 2.4
Total Solids	g Kg^−1^	17.6 ± 1.3	21.7 ± 1.2	26.6 ± 2.6	29.3 ± 1.4	38.2 ± 5.0
Volatile Solids	g Kg^−1^	10.2 ± 1.0	13.7 ± 1.2	18.9 ± 2.5	21.1 ± 1.2	29.1 ± 4.6
COD_T_	g O_2_ L^−1^	17.2 ± 1.0	24.1 ± 3.5	33.2 ± 2.5	36.6 ± 3.8	54.8 ± 8.34
CODs	g O_2_ L^−1^	3.9 ± 1.0	5.5 ± 0.5	8.6 ± 2.9	7.2 ± 0.1	19.3 ± 6.5
BOD_5_	g O_2_ L^−1^	2.0 ± 0.6	3.3 ± 1.1	4.4 ± 0.1	4.9 ± 0.1	12.9 ± 0.1
Total Nitrogen	g ^L-1^	3.2 ± 0.3	4.5 ± 0.6	5.8 ± 0.3	6.7 ± 0.6	8.0 ± 0.8
NH_4_^+^-N	g ^L-1^	2.2 ± 0.2	2.9 ± 0.2	2.8 ± 0.2	3.0 ± 0.2	3.7 ± 0.4
NH_3_-N	g ^L-1^	0.1 ± 0.1	0.3 ± 0.1	1.1 ± 0.2	1.4 ± 0.2	1.6 ± 0.3
Acetic acid	mg L-1	376 ± 301	646 ± 168	1647 ± 858	1836 ± 919	3117 ± 1526
Propionic acid	mg L-1	35 ± 10	173 ± 66	629 ± 614	713 ± 623	1582 ± 521

*n.d* non detected

Biogas and methane production, biogas characteristics, specific biogas and methane productions and sludge characteristics from the anaerobic digestion of *Spirulina* obtained with the five organic loading rate tested. Shown are the mean values of each period with their respective standard deviation

**Fig. 1:**
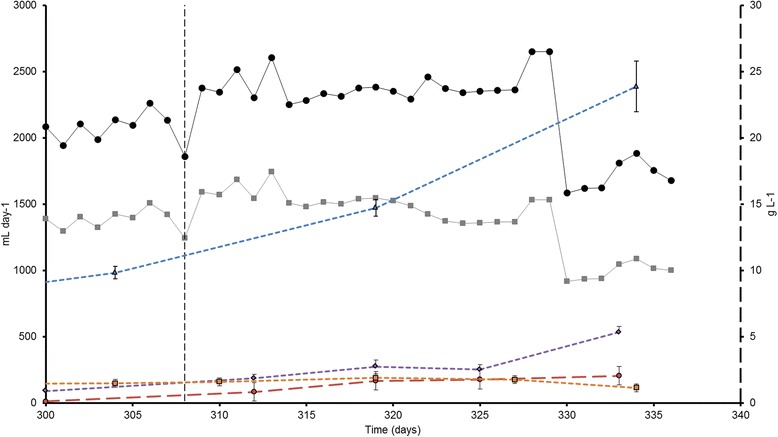
Biogas, methane production and sludge characteristics. Left axis: daily biogas (Black-●) and methane (Gray-■) production normalized to standard temperature and pressure conditions (20 °C; 0 atm); Right axis: Soluble COD (Blue- ∆); acetic acid (Purple- ◊); propionic acid (Red-o), NH_3_-N (Orange-■) obtained from the anaerobic digestion of *Spirulina.* Vertical dashed line indicates a change in the organic loading rate from 4.0 g *Spirulina* L^−1^ day^−1^ to 5.0 g *Spirulina* L^−1^ day^−1^

The increment in biogas production was not completely proportional to the loading rate (Table 1). Apparently, at higher loading rates digestion of the algal biomass was no longer complete, which eventually led to substrate overload causing reactor failure (Fig. 1). This was apparent from: (i) the drop in biogas production at the end of period V (Fig. 1), (ii) the decreasing methane content of the biogas at high loading rate and (iii) the increase in all the parameters related to organic matter, TS, VS, total and soluble chemical oxygen demand (COD) and five day biological oxygen demand (BOD_5_) (Table 1). This accumulation of organic matter was especially acute during period V with a 30 % increase for TS, 37 % for VS, 50 % for total organic matter (COD_T_), and 163 % for BOD_5_ compared to period IV. Soluble organic matter, acetic acid, propionic acid and ammonia also accumulated in period V (Fig. 1, Table 1). *Spirulina* is a protein rich substrate [19], therefore its nitrogen content is high, which can explain the observed accumulation of total nitrogen (Table 1). The degradation of proteins leads to the production of ammonium nitrogen (NH_4_-N) which increased gradually from 2.2 g L^−1^ in period P-I, to 3.7 g L^−1^ in period P-V while, at the same time ammonia nitrogen (NH_3_-N) increased from 0.1 g L^−1^ to 1.6 g L^−1^ (Table 1, Fig. 1). This increase in NH_3_ can be attributed both to the increase in substrate concentration and, according to the equation of Anthonisen et al*.* [21], to the increase of pH from 7.5 to 8.6. Methanogens are sensitive to ammonia [22] and the accumulation of this compound can lead to digester failure. The reported levels of free ammonia nitrogen in period V are high and comparable to other reported levels that caused reactor inhibition [22–25]. The accumulation of non-degraded biomass and VFAs along with the relatively high total nitrogen (8.0 g L^−1^) and free ammonia nitrogen (1.6 g L^−1^) concentrations led to the reduction of the biogas production from 2651 mL day^−1^ on day 329 to 1586 mL day^−1^ on day 330 (Fig. 1) which is consistent with a substrate overload in the digester.

One of the main bottlenecks perceived for the anaerobic digestion of microalgal biomass is low biodegradability, which can result in low methane yields [26]. To overcome this problem long HRT need to be applied in order to increase the residence time allowing the substrate to be further hydrolyzed [8]. However, this on the long run can have a negative effect on the biogas production as accumulation of inhibitory substances such as ammonia can occur [8, 27]. Our results show that at 20 days HRT an ORL of 4.0 g *Spirulina* L^−1^ day^−1^ (period IV) results in a good compromise between an optimal methane yield and accumulation of toxic compounds. At this OLR, the specific biogas production (SBP) per gram of *Spirulina* was 350 mL biogas with 68 % methane content (Table 1). Moreover, the highest biodegradability, 42 %, was obtained, and no accumulation of inhibitory substances occurred in this period. Both values, SPB and biodegradability are similar to those observed in other studies [5, 7, 10].

### Metagenome analysis of the anaerobic digester community

DNA was extracted at the end of period V (day 336) from the sludge of the *Spirulina* digester and sequenced on a 318^TM^ Chip with the Ion Torrent Personal Genome Machine (PGM) platform. Obtained sequence reads were quality filtered and trimmed (Table 2). The remaining reads were either assembled into contigs or analyzed directly with the MG-Rast metagenome pipeline.

**Table 2:** Sequencing statistics

		Bases		Reads	Mean read length		GC %
Sequencing data							
PGM raw data		1 GB		5630598	155 (bp)		38
Post trimmomatic data		974 MB		5240830	185 (bp)		38
Assembled contigs							
Assemblies	# Submitted reads	Minimum read length	# Contigs	# Contigs >500 bp	N50 contig size	Mean contig size	Largest contig size
Assembly A^a^	5240830	50 (bp)	54246	21998	3810 (bp)	1807 (bp)	171327 (bp)
Assembly B^a^	5240830	50 (bp)	278958	30987	1226 (bp)	1115 (bp)	18073 (bp)
Assembly C^a^	1984110	220 (bp)	27994	14915	4380 (bp)	1899 (bp)	75139 (bp)

Dataset-1 sequencing data and assemblies statistics

^a^See Material & Methods for details about assembly settings

#### Binning and 16S rDNA taxonomy analysis of assembled contigs

Three different assemblies were used for the detection of ribosomal 16S genes to taxonomically characterize the microbial community (see Material and Methods for details). Assembly A produced the largest contig, 171,327 bp. Due to the stringent settings, assembly B produced a higher number of shorter contigs (N50 value of 1226 bp), while assembly C (only reads with a minimum read length of 220 bp were assembled) produced contigs with the longest mean size and the longest N50 value, 4380 bp (Table 2). The same 16S rRNA gene sequences were all assembled in each of the three assemblies. However, for some of the dominant taxa, the length of assembled 16S rRNA gene fragments were different between the three assemblies. Assembly A produced the longest contig for the Methanosarcinales 16S rRNA gene, assembly B yielded the longest Anaerolineales 16S sequence, and in assembly C the longest 16S sequences for Bacteroidales and Clostridiales were obtained. The 16S sequences of Methanomicrobiales and Thermotogales were assembled to approximately equal lengths in all three assemblies. The 16S genes of Flavobacteriales and Lactobacillales were better assembled in assemblies A and C than in assembly B.

Although Assembly C yielded the longest average 16S sequences, the length of the archaeal 16S sequences was greater in assembly A. An MG-Rast analysis of the reads submitted to assemblies A and C, respectively, revealed that the percentage of reads assigned to Firmicutes was markedly higher in the reads submitted to assembly C. This suggests that, with the removal of reads shorter than 220 bp from the read set submitted to assembly A in order to obtain the read set for assembly C, Firmicutes sequences were enriched in the latter.

Contigs from assembly A were considered to better represent the diversity in the biogas reactor and were selected for binning using the Metawatt v1.7 pipeline to investigate the most abundant populations of the microbial consortium in more detail. From the 113 obtained bins, after manual selection and curation, 10 remained which displayed characteristic tetranucleotide frequencies, assembly coverages and consistent phylogenetic signature and together accounted for almost 80 % of the total sequence data (Table 3; Additional file 1: Figure S1). As was observed in other anaerobic digesters, populations affiliated with Firmicutes were most abundant, and constituted 38 % of the total community [13–17, 28], followed by Bacteroidetes (abundance approx. 13 %), Chloroflexi (8 %) and Thermotogae (7 %). Contigs affiliated with Euryarchaeota comprised a single bin and represented only 3.5 % of the total abundance. Two bins of unknown taxonomic origin accounted for 10 % of the sequenced data (Table 3).

**Table 3:** Characteristics and taxonomical classification of selected microbial bins

Bin characteristics									16S rDNA taxonomical classification
Bin	Contigs (#)	Size (Mb)	N50 contig length (Kb)	GC (%)	Cov (X)	tRNA (#)	Conserved genes (#)	Abun (%)	
A	443	1.87	9.8	32.0	66.8	21	113/139	14.9	*Un. Tissierella*
B	489	2.58	17.2	29.8	42.4	19	105/139	13.1	*Un. Clostridiales*
C	443	2.40	13.0	31.9	36.6	21	79/139	10.5	*Un. Clostridiales*
D	468	3.37	22.5	42.0	22.3	77	103/139	9.0	*Proteiniphilum*
E	384	1.91	16.2	47.3	33.3	49	92/139	7.6	*Un. Anaerolineaceae*
F	397	2.44	27.7	31.1	23.4	49	106/139	6.9	*Un. Thermotogaceae*
G	225	2.53	39.5	51.6	21.8	71	164/139	6.5	*Unknown*
H	879	3.05	7.9	37.8	11.5	44	82/139	4.2	*Un. Bacteroidetes*
I	4267	4.10	1.6	49.2	7.1	70	75/139	3.5	*Unknown*
J	8444	5.11	0.7	55.5	5.6	31	161/139	3.5	*Un. Methanomicrobia*

*Cov* Coverage

*Abun* Abundance

*Un* Uncultured

Characteristics and 16S rDNA taxonomical classification of the 10 selected bins obtained from the *Spirulina* metagenome

**Fig. 2:**
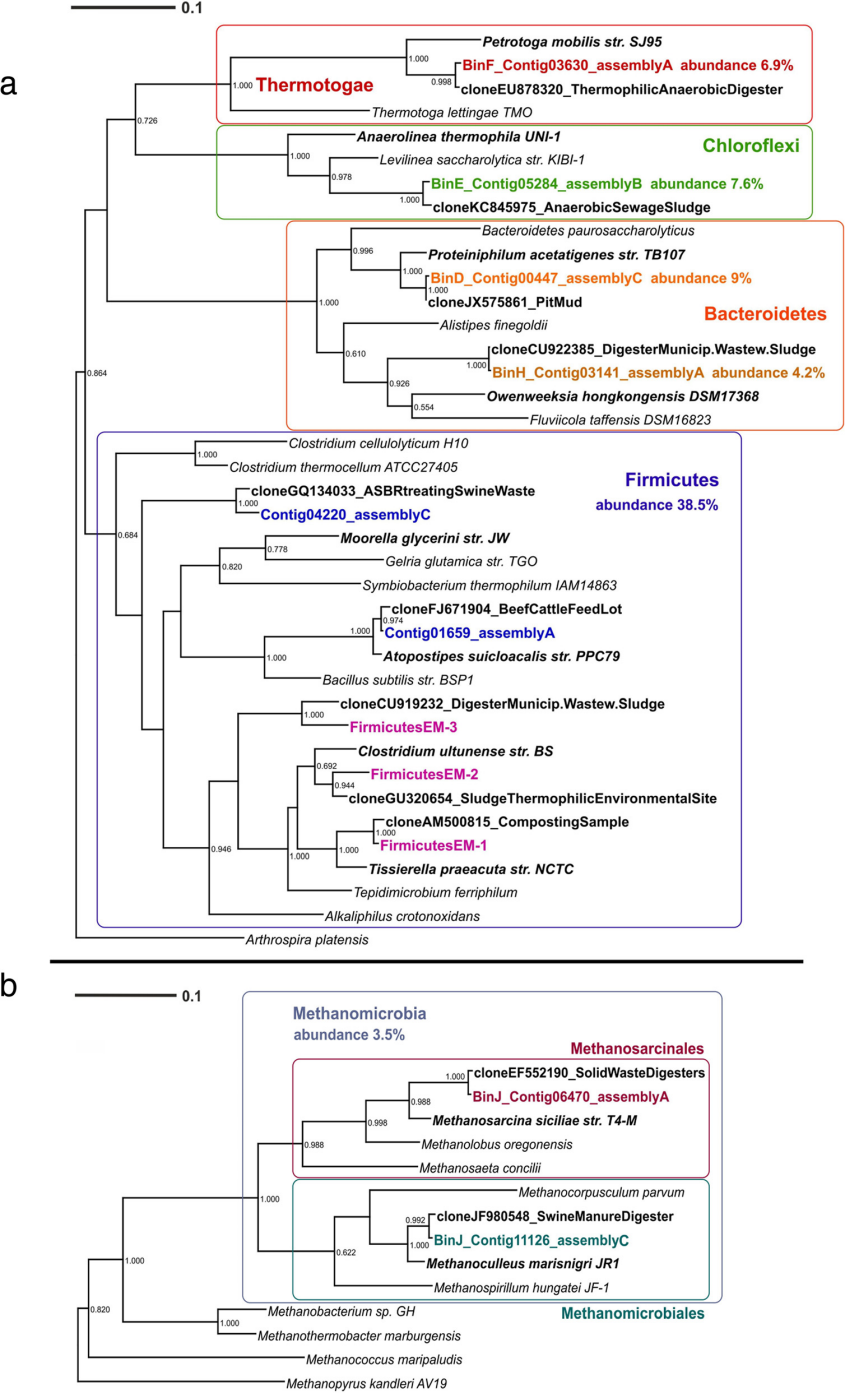
16S rDNA bacterial and archaeal phylogenetic trees. Phylogenetic trees of (**a**) Bacterial and (**b**) Archaeal 16S rDNA sequences. Although the binning was only performed with contigs of assembly A, most of the binned 16S rDNA sequences were also assembled in assemblies B and C. If so, for the tree the longest assembled sequence was chosen. 16S rDNA sequences not assembled in assembly A, but in B or C, or detected by EMIRGE are also included. Colored: sequences obtained from metagenomic reads. Assignment to metawatt bins is indicated if applicable. Reference sequences in bold: top hits in blast search against NCBI non-redundant nucleotide collection, *bold + italics*: top hits in blast search against NCBI reference RNA sequences. Additional reference sequences in the Bacteria tree represent genera detected in other anaerobic digesters. 16S rDNA sequences of *Arthrospira platensis* and *Methanopyrus kandleri* were chosen as outgroups, respectively. Bootstrap values at nodes are obtained from 500 replicates and are only shown for branches with at least 50 % support (values > 0.499). The scale bar represents 0.1 nucleotide substitutions per site. Accession numbers of reference sequences are available in Additional file 2: Table S7

Ribosomal rRNA gene sequences corresponding to 8 of the 10 bins were identified among the contigs of the three different assemblies and/or recovered independently by iterative read mapping with EMIRGE (Fig. 2a, Additional file 2: Table S1). Assembled 16S rRNA sequences identical or highly similar to the EMIRGE sequences were not detected. However, 16S sequences similar to EM-1 (up to 97.74 % for aligned part of 752 bp) and EM-2 (up to 95.04 % for 1269 bp aligned) were found among unbinned contigs of assembly A and/or contigs of assemblies B and C (Additional file 1: Figure S2). No assembled 16S sequence similar to EM-3 was found. Unfortunately, the three 16S sequences obtained by EMIRGE and affiliated with Firmicutes (EM-1, EM-2 and EM-3) could not be assigned conclusively to any of the three Firmicutes bins (A, B and C). However, based on comparison of sequencing coverage between the recovered EMIRGE 16S sequences and the contigs in the Firmicutes bins it was most likely that the dominant organism, Bin-A, 14.9 % abundance, (Table 3) was most closely related to *Tissierella praeacuta* (EM-1, Fig. 2a). Bins B and C were assigned to uncultured Clostridiales (Table 3). The 16S rDNA sequence classified as *Atopostipes* (contig01659 of assembly A) was assembled well in all three assemblies, yet it could not be linked to any of the bins. Based on the low sequencing coverage of this 16S sequence, the population was probably of relatively low abundance and poorly assembled (Additional file 2: Table S2).

As in a previous study of anaerobic digestion in which a proteinaceous substrate was used, Clostridiales were the most abundant bacterial order [28]. The genus *Tissierella* has already been detected in other anaerobic digesters [14, 29–31], but the specific function of members of this taxon in anaerobic digesters is still not clear. However, members of this genus are known to require the presence of certain amino acids and formate for growth and they seem to be unable to utilize carbohydrates such as glucose, cellobiose or xylose [32] which is in accordance to the type of substrate used in our experiment. *Proteiniphilium*, (Bin-D, Table 3) a member of the Bacteroidales that utilizes peptone and is unable to grow on carbohydrates [33], was previously identified in several biogas studies [12, 14, 29, 34]. Anaerolineales and Thermotogales were also identified in other biogas reactors but in much lower abundance [15, 35, 36]. Their function in anaerobic digestion is not clear yet, however, their relatively high abundance when compared with other anaerobic reactors could be explained by the fact that they are known to utilize proteins as substrate [37, 38]. Therefore they might play an important role in the degradation of protein-rich *Spirulina*. The 16S sequence in Bin-D (contig_00447 of assembly C) shows 94.4 % similarity to *Proteiniphilum acetatigenes* str. TB107 (Porphyromonadaceae), with differing lengths of homopolymer stretches accounting for 1.5 % of the differences. The most similar (99.3 %) described sequence to contig_00447 is the Porphyromonadaceae clone JX575861.1. This clone sequence shows 95.5 % similarity to the 16S gene of *Proteiniphilum acetatigenes*, which supports the classification of Bin-D as an unknown *Proteiniphilum*.

Among the Archaea, we identified one bin, Bin-J, with 3.5 % of abundance, for which the 16S rDNA fragments were classified as uncultured Methanomicrobia (Table 3). Methanomicrobiales were also identified as the most abundant methanogens in an anaerobic reactor fed exclusively with casein [28, 39]. A closer look at the 16S rDNA phylogeny (Fig. 2b and Additional file 2: Table S1) suggests the possibility that two methanogenic populations may have been binned together, one related to Methanomicrobiales and one to Methanosarcinales. Members of these orders, which are able use H_2_, CO_2_, formate and acetate as their C source [14, 40], are frequently encountered in anaerobic digesters [13, 41]. Formate and acetate are both fermentation products of Clostridiales and Anaerolineales [32, 37], both populations present in high abundance in our experiment (Table 3).

#### Comparison of two metagenomes by short single read analysis

In parallel to this genome-focused analysis of assembled contigs, the effect of substrate on both taxonomic composition and presence of functional genes was also studied at the level of individual reads, by comparing the unassembled reads from our study to a publicly available metagenome from a biogas plant using the MG-Rast platform. Sequencing dataset *Spirulina*-S1, was obtained from our anaerobic reactor fed with *Spirulina*, a protein rich substrate, while the second sequencing dataset, Maize-Rye (M-R), originated from a biogas plant fed with a mixture of mainly cellulose rich substrates [13].

##### Effect on the microbial community composition

The general taxonomic composition did not appear to depend on the type of substrate used. Based on the MG-Rast M5NR analysis of the metagenomic reads, bacteria clearly dominated in both datasets while Archaea represented less than 10 % in the M-R dataset and merely 3 % in the *Spirulina*-S1 dataset. Among bacteria, Firmicutes, Bacteroidetes and Proteobacteria dominated in both sets, but differences could be seen in the abundances of other phyla. For example, hits in Thermotogae were more abundant in the protein rich substrate digester data (5.4 %) compared to 2 % in the cellulose rich substrate digester dataset. A similar result was observed with Chloroflexi, 2.7 % in *Spirulina*-S1 when compared to the M-R dataset, 1.3 %. On the other hand, within the Archaea such a variety at phylum level was not seen, and as expected, Euryarchaeota dominated with over 90 % of all Archaea in both datasets.

**Table 4:** Comparison of identified COG and specific Pfams

	*Spirulina*-S1 dataset		Maize-Rye dataset	
Initial # reads	1019333		1019333	
Initial # ORF	6115998		6115998	
	Hits	%	Hits	%
COG				
Total # hits	206325		323947	
Information storage and processing	47949	23.2^*a*^	85124	26.3^*a*^
Cellular processes and signaling	37543	18.2^*a*^	59222	18.3^*a*^
Metabolism	89072	43.2^*a*^	124053	38.3^*a*^
Poorly characterized	31761	15.4^*a*^	55548	17.1^*a*^
Ratio AaM/CM^b^	1.27		0.91	
PFAMs				
Total identified PFAMs	1132766		2205177	
Cellulose degradation PFAMs	1881	0.17^c^	6554	0.30^c^
Amino acid degradation PFAMs	10628	0.94^c^	16052	0.73^c^
Protein degradation PFAMs	7148	0.63^c^	11498	0.52^c^
Ratio proteases/cellulases (P/C)	3.80		1.75	
Ratio amino acid/cellulases (A/C)	5.65		2.45	

^a^% of total identified COGs

^b^“Amino acid transport and metabolism”/“Carbohydrate transport and Metabolism”

^c^% of total identified PFAMs

Comparison of the COG categories obtained with the MG-Rast platform (E-value 1e^−5^; min 60 % identity; 15 bp min length) and the selected Pfams obtained with the Hmmscan (E-value cutoff 1.0)

**Fig. 3:**
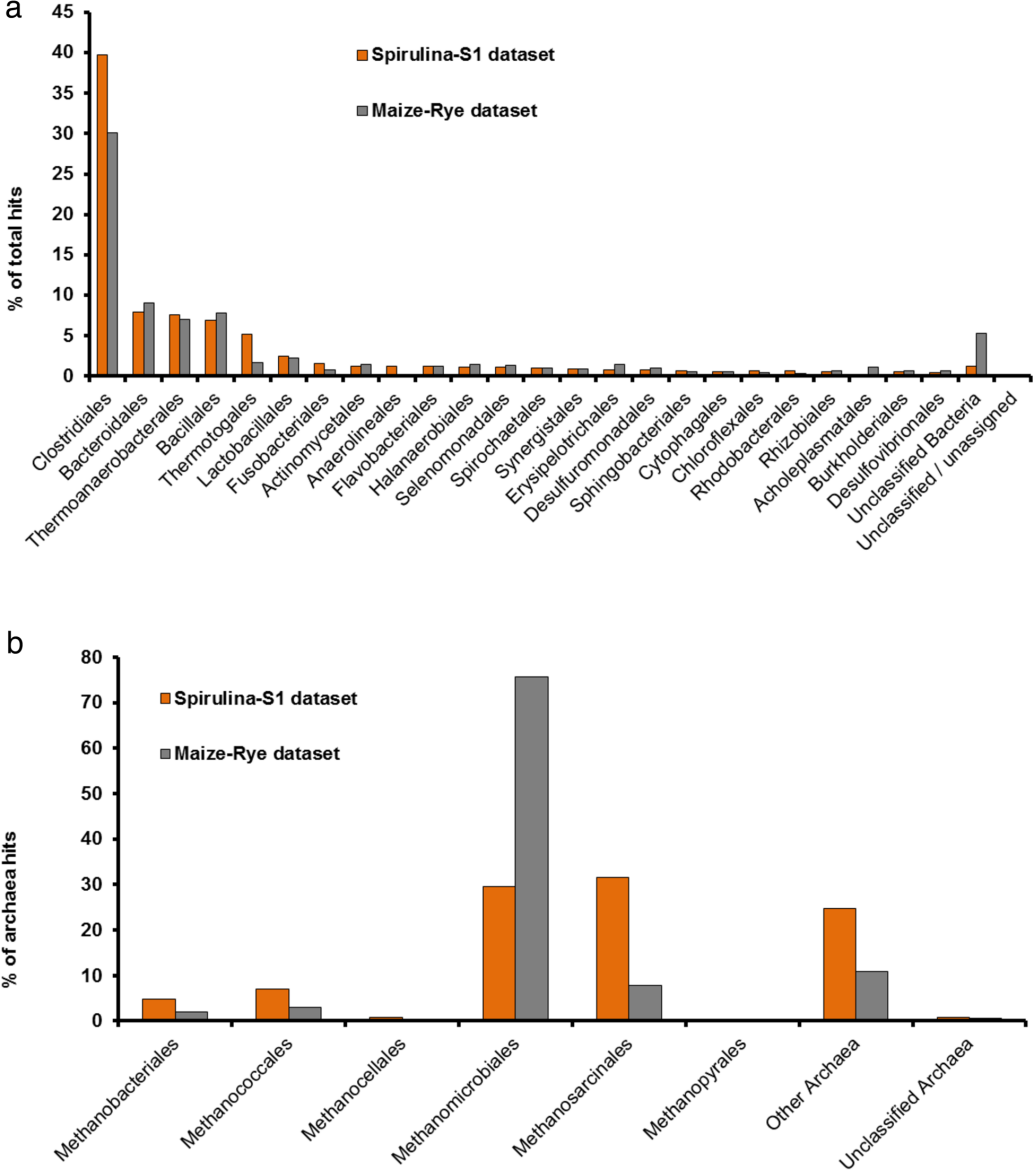
Comparison of the taxonomic classification of *Spirulina*-S1 and M-R reads at order level. Percentage of the total taxonomic assigned reads of each dataset obtained with MG-Rast M5NR representative hit tool (E-value 1e^−5^; min 60 % identity; 15 bp min length). **a** Bacterial orders with the 20 most abundant assigned read hits and (**b**) all Archaeal orders with assigned hits from the metagenomic reads

Among the Bacteria, Clostridiales dominated in both datasets with 40 and 30 % of the total assigned reads in the *Spirulina*-S1 dataset and the M-R dataset respectively, followed by Bacteroidales, Thermoanaerobacterales and Bacillales (Fig. 3a). In both datasets, M-R and *Spirulina*-S1, the genus to which most of the reads were assigned was *Clostridium* (18 and 14 % of recruited reads respectively) (Additional file 2: Table S3). Furthermore, as was also found by Kovács et al. [28], who used casein and pig blood as substrate, a relatively high number of reads in the *Spirulina*-S1 dataset (almost 40,000 reads – 6 % of the total hits) were most similar to members of the genus *Alkaliphilus*, which is in contrast to the M-R dataset, where only about 2 % of the reads were assigned to this genus (18,000 hits). Bacteroides, recruiting 7.5 % of the hits in the M-R dataset accounted for 4.1 % in the *Spirulina*-S1 dataset. Among other substrates, Bacteroides are known to utilize cellobiose and xylose [34], both absent in *Spirulina*, which could explain their lower abundance in our reactor. Interestingly, *Candidatus Cloacamonas*, which accounted for 4,1 % of the bacteria M-R reads, 36,032 hits, recruited less than 0.1 % of the hits in the *Spirulina*-S1 dataset (280 hits) (Additional file 2: Table S3). This bacterium was also present in high abundance at the initial adaptation period of two biogas reactors fed with casein and pig blood and its detection was not possible after 12 weeks of substrate adaptation, which might indicate that it cannot survive without a source of carbohydrates [28]. Major differences regarding bacterial taxa were also seen for Thermotogales and Anaerolineales which were considerably more abundant in the dataset from the protein rich substrate digester, with 5 and 1 % respectively when compared to the dataset from the cellulose rich substrate biogas plant, 1.7 and 0.1 % respectively (Fig. 3a). These two orders are known to include bacteria which utilize proteins as substrate [36, 37, 39].

Among the archaeal orders, Methanomicrobiales clearly dominated in the M-R dataset, recruiting almost 75 % of the Archaeal hits. In the *Spirulina*-S1 dataset, the combined presence of Methanosarcinales (31 % of the hits) and Methanomicrobiales (29 % of the hits) was observed (Fig. 3b; Additional file 2: Table S3), confirming the analysis of the assembled 16S rDNA sequences (Fig. 2b). Methanosarcinales are known to be able to use acetate, H_2_ and CO_2_ as substrate [40], which are the main fermentation products of *Tissierella* and other Clostridia [32], and they are usually dominant in reactors where VFAs and NH_3_ are present in high concentration as in our case (Fig. 1) [42, 43]. On the other hand, Methanomicrobiales do not use acetate but can grow on H_2_, CO_2_ and formate [44], the latter a common fermentation product of bacteria of the phylum Chloroflexi [37] which in our dataset represented almost 8 % of the abundance (Table 3). The presence of two methanogenic populations in an anaerobic digestor is quite common and has also been observed in several other studies. For example Ziganshin et al*.* [45] observed the presence of *Methanoculleus* and *Methanosaeta* in reactors fed with cattle manure and dried distillers grains, and of *Methanosarcina* and *Methanoculleus* in reactors treating maize straw and cattle manure, while Li et al*.* [15] also detected the same two groups of methanogens in a reactor treating multiple substrates (chicken waste, pig manure and excess sludge). This presence of different methanogens detected in such a broad range of substrates might indicate that, rather than the type of used substrate, the characteristics of the sludge (NH_3_, VFAs, temperature, pH, etc.) and the initial type of inoculum (wastewater, manure, etc.) determine which Archaea thrive in anaerobic digesters.

In order to investigate whether the diversity of the species community was affected by the used substrates, the species diversities in both datasets, were analyzed by means of Lorenz curves and Simpson’s diversity index (SDI) [46–48] and compared with each other. The mixture of substrates used in the biogas plant (maize silage, 63 %; green rye, 35 % and chicken manure, ~2 %), might lead to a higher bacterial diversity than in the reactor solely fed with *Spirulina*. However, Lorenz curves, describing population evenness, of both datasets were similar (Additional file 1: Figure S3a), as well as Simpson’s diversity index values (SDI = 0.0078 for the *Spirulina*-S1 dataset and SDI = 0.0062 for the M-R dataset; where SDI = 1 indicates low diversity and SDI = 0 indicates high diversity). The similarity in evenness and diversity in both bacterial populations can be explained by the fact that *Spirulina* as such is not a “simple” substrate, as would be glucose, starch or glycerol, and therefore needs a microbial population with a certain complexity to be fully digested. Apparently, the differences between the substrate types (complex mono-substrate or substrate mixture) used in the two compared systems did not affect the diversity of the whole population, yet rather the abundance of certain bacterial taxa (Fig. 3).

For Archaea, on the other hand, Lorenz curves and SDI indicated a difference in their diversity in the two studied datasets. The archaeal population in the *Spirulina* reactor was more even than the archaeal community in the maize-rye biogas plant (Additional file 1: Figure S3b). Given the same species richness, a more even population is also more diverse. Indeed, by calculating the SDI a higher diversity for the archaeal community in the *Spirulina* reactor was observed (SDI = 0.0761) than in the M-R biogas plant (SDI = 0.3695). The lower SDI for the Archaea in the M-R dataset is best explained by the clear dominance of *Methanoculleus* in the archaeal reads of this dataset (Fig. 3b).

Taken together, the results suggest that the type of substrate used in anaerobic digestion mainly affects the bacterial composition, to some extent, at low taxonomic levels, especially at genus and species level. Proteolytic Bacteria were probably present in all dominant phyla of the *Spirulina*-S1 digester, whereas in the Maize-Rye biogas plant, cellulolytic Bacteria were dominant. Regarding the Archaea, an influence of the substrate on their presence and composition is not as clear as for the Bacteria. This could be explained by the fact that the Archaea perform the final step in the process of anaerobic digestion, and their presence is probably more dependent on the population composition of the primary substrate degraders (Bacteria), of their metabolic products, the presence or absence of inhibitory compounds and the origin of the inoculum, rather than on the substrate itself.

##### Effect on the abundance of functional genes

Both datasets, *Spirulina*-S1 and M-R were compared at functional level with two approaches, MG-Rast’s COG comparison, and identification of specific protein domains (Pfam) related to the cellulose degradation pathway and to protein and amino acid degradation (Table 4).

**Fig. 4:**
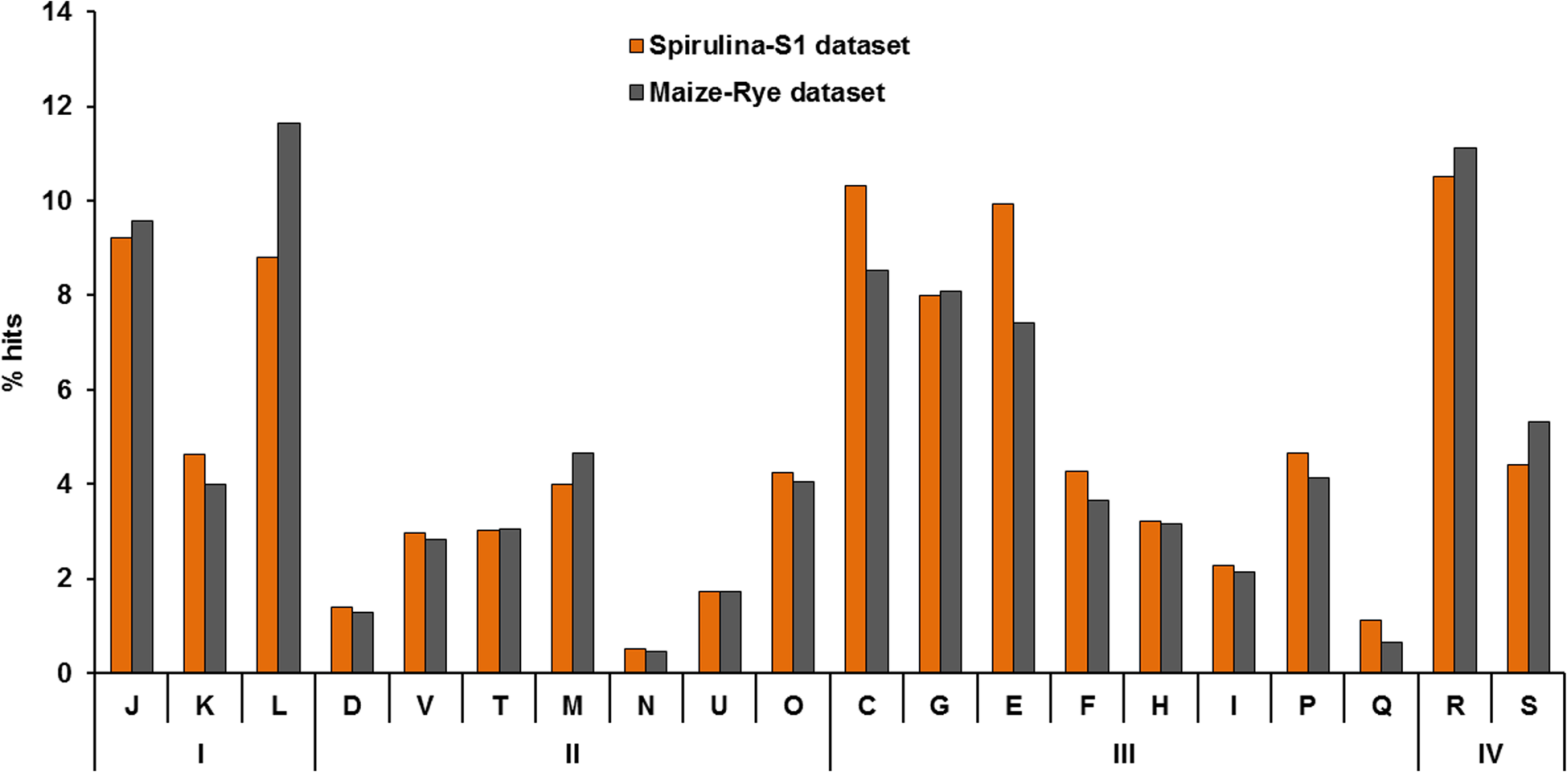
COG functional hierarchical classifications. Comparison of the COG classified reads of the *Spirulina*-S1 and the M-R metagenomes obtained with the MG-Rast metagenome analyzer (E-value 1e^−5^; min 60 % identity; 15 bp min length). Only those categories with hits are represented. X axis: (I) Information storage and processing: J, translation, ribosomal structure and biogenesis; K, transcription; L, replication, recombination and repair; (II) Cellular processes and signaling: D, cell cycle control, cell division, chromosome partitioning; M, cell wall/membrane/envelope biogenesis; N, cell motility; O, posttranslational modification, protein turnover, chaperones; T, signal transduction mechanisms; U, intracellular trafficking, secretion and vesicular transport; V, defense mechanisms; (III) Metabolism: C, energy production and conversion; E, amino acid transport and metabolism; F, nucleotide transport and metabolism; G, carbohydrate transport and metabolism; H, coenzyme transport and metabolism; I, lipid transport and metabolism; P, inorganic ion transport and metabolism; Q, secondary metabolites biosynthesis, transport and catabolism; (IV) Poorly characterized: R, general function prediction only; S, function unknown

Due to the different types of substrates used, it was expected to see differences regarding the COGs related to amino acids and protein metabolism. However, with respect to the detected functional genes, the differences between both datasets were minor (Fig. 4). The highest difference was observed in category L, “Replication, recombination and repair”, which represented 8.8 % of the hits in the *Spirulina*-S1 dataset and 11.6 % in the M-R dataset and contains COGs related to groups of genes which participate in the replication process of the microbial community. This difference in abundance was significant (*p* value = 0.023) and could be explained by the fact that at the time of sampling of the *Spirulina*-S1 dataset, day 336, the reactor was suffering from substrate overload which resulted in an accumulation of toxic compounds (NH_3_, VFAs) that hindered the correct function of the bacteria and reduced the daily biogas production (Fig. 1). The COGs related to category E, “Amino acid transport and metabolism” were slightly more abundant in the protein rich dataset (9.9 %), than in the cellulose rich dataset (7.4 %) (*p* value = 0.067). The higher relative abundance of COGs from category E in the *Spirulina*-S1 dataset could be attributed to the type of substrate used. However, as most of the COGs from this category represent enzymes involved in general metabolic processes, further research must be conducted in order to fully verify this result. On the other hand, and contrary to what was expected, no significant differences (*p* value = 0.578) could be seen for the COGs from “Carbohydrate transport and metabolism” (category G) in both datasets (Fig. 4). However, it should be noted that, in category G, important differences could be seen among those COGs directly related to the degradation of cellulose, xylanose and other complex sugars. For example, COG3664 a “Beta-xylosidase” represented 0.10 % in the M-R dataset and was not detected in the *Spirulina*-S1 dataset. Similarly, COG2160, an “L-arabinose isomerase”, was almost absent in the *Spirulina*-S1 dataset (0.005 % of all assigned hits) but represented almost 0.05 % in the M-R dataset, likewise COG3693 a “Beta-1,4-xylanase” and COG0366 a “Glycosidase” were both significantly less abundant in the *Spirulina*-S1 dataset. This difference in the abundance of COGs correlates with the type of substrate used, *Spirulina*, which has a low content of complex sugars [19].

To corroborate the results obtained with the COGs analysis, specific protein domains (Pfam) related to cellulose degradation and to protein and amino acid degradation were searched for in both datasets (Additional file 2: Tables S4, S5 and S6). Pfam domains associated with cellulose summed up to 0.30 % of the total identified Pfams in the M-R dataset compared to 0.17 % in the *Spirulina*-S1 dataset (Table 4). The proteases related Pfams were slightly more abundant in the *Spirulina*-S1 dataset with 0.63 % of all identified Pfams compared to 0.52 % in the M-R dataset (Table 4). Also the amino acid degradation Pfams were more abundant in the *Spirulina*-S1 reads (0.95 % of all the Pfams) than in the M-R reads (0.74 %).

Two Pfam ratios, Proteases to Cellulases ratio (P/C ratio), and Amino acid to Cellulase ratio (Aa/C) were also calculated to determine the relative abundance of each group. The P/C ratio in the *Spirulina*-S1 dataset, 3.80, was double the P/C ratio in the M-R dataset, 1.75, and a comparable result was obtained with the Aa/C ratios, 5.65 for the *Spirulina*-S1 and 2.45 for the M-R dataset (Table 4). When a COG “Amino acid transport and metabolism” to “Carbohydrate transport and metabolism” ratio was calculated (AaM/CM), the result was similar, 1.27 for the *Spirulina*-S1 dataset and 0.91 for the M-R dataset (Table 4).

The differences in the obtained P/C, Aa/C and AaM/CM ratios, plus the higher abundance of amino acid metabolism related COGs suggest that the microbial community in the *Spirulina* reactor adapted to the type of substrate degraded, protein rich with low presence of cellulose. However, since substrates from both reactors were not either pure carbohydrates or proteins, but consisted of both in different proportions, further studies are necessary to determine whether the observed differences in the presence of functional genes would become more distinct for longer run times of the *Spirulina* digester, or if the microbial community was already at functional equilibrium when it was sampled for the metagenome analysis presented here.

#### Binning of contigs vs classification of single short reads

In this work, the same metagenome has been analyzed by two different approaches, assembly of reads into contigs followed by a binning strategy combined with a 16S rDNA analysis, and blasting of single reads against a general nucleotide database. Despite the differences in methodology, the results obtained with both approaches were consistent (Table 3; Fig. 3). The first advantage of using the assembly/binning approach is that the obtained bins form a provisional whole genome sequence of the most abundant organisms (Table 3). This way, the function of individual populations can be inferred directly. The second advantage is that taxonomical classification of contigs is more reliable than the classification of short reads. In contrast, the main advantage of using MG-Rast classification of short reads is that it is more sensitive because functions can be inferred from the presence of non-redundant reads originating from minor community members that remain unassembled. Since ~80 % of the sequenced reads could be mapped to the 8 bins corresponding to the 8 most abundant populations, approximately 20 % of the microbial community was below the detection limit of the assembly/binning approach.

## Conclusions

The results presented in this study suggest that *Spirulina* is a suitable substrate for the production of biogas with a mean production of 350 ml of biogas per gram of substrate. As in previous studies, the most abundant Bacterial populations making up the consortium performing digestion belonged to Clostridia and Bacteroidetes whereas the most abundant Archaeal populations were affiliated with Methanomicrobiales and Methanosarcinales. The microbial community present in the anaerobic digester was well adapted to the type of substrate used, based on taxonomic and functional inferences. Taxonomic analysis of assembled contigs with a binning approach produced results consistent with the classification of short single reads.

## Methods

### Bioreactor set-up

One 2.0 L semi-continuous stirred tank reactor (S-CSTR) with a working volume of 1.5 L, operated at 37 °C with 20 days hydraulic retention time (HRT) was set up to study the anaerobic digestion of freeze dried *Spirulina*. The overall experiment lasted 440 days which included a 33 days adaptation to *Spirulina* and a 71 days start-up period. The remaining 336 days were divided into 5 periods (P-I to P-V) in which the organic loading rate (OLR) was gradually increased from 1.0 g to 5.0 g *Spirulina* L^−1^ day^−1^ (dry weight). The inoculum was obtained from a local wastewater treatment plant (Heepen Klaerwerk, Bielefeld, Germany) and the substrate, freeze dried *Spirulina,* was acquired from Sonnenmacht GmbH (Germany). According to the manufacturer, the *Spirulina* biomass contains 64 % proteins, 3.5 % carbohydrates and 6 % of lipids. Biogas production was measured with an on-line Milligascounter MGC-1 equipped with the Rigamo software v3.0 (Ritter Engineering, Germany) and normalized to standard conditions (0 °C; 1.0 atm). pH and redox potential were monitored, but not controlled, with Mettler Toledo pH (HA405-DPA-SC-S8/225) and redox (Pt4805- DPA-SC-S8/225) probes (Mettler Toledo GmbH, Germany). Mesophilic conditions were obtained with a Pt-1000 temperature sensor and a heater. In order to avoid rupture of the bacterial granules, constant stirring was performed with a floating magnet (Fisher Scientific GmbH, Germany). Daily purge and feed were performed manually with a syringe. Before purging, the biomass was settled by stopping the stirring for at least 30 min. Periodically the purged sludge was sampled for analysis; in that case the stirring was not stopped. The medium used to dissolve the freeze dried *Spirulina* for dosing at the desired OLR was modified after Vidal et al*.* [49] excluding the NH_4_Cl. The *Spirulina* mixture was prepared freshly once per day.

### Analytical methods

The performance of the laboratory digester was continuously monitored by the on-site pH probe, the biogas measurement device and by periodical analysis of alkalinity. Carbon dioxide content of the biogas was determined daily by bubbling the produced biogas through an alkaline solution (KOH 50 g L^−1^). Biogas composition was determined once a week by means of a Shimadzu GC-2010 plus Gas Chromatograph (Shimadzu Corp, Japan) equipped with an Agilent GS-Gaspro capillary column (part # 113–4362) (Agilent Technologies, USA). Samples for biogas quality and composition were obtained using an airtight syringe. If biogas composition was not analyzed immediately, samples were kept in gas-tight vacutaniers (BD-Plymouth, UK). Analyses to characterize the liquid effluent were carried out periodically. Total solids (TS) and volatile solids (VS) were analyzed once a week following the APHA standard methods [50]. Five day biological oxygen demand (BOD_5_) was analyzed with a WTW Oxitop® according to the APHA 2005 5210D procedure. Alkalinity, total and soluble chemical oxygen demand (COD_T_ and COD_S_), total nitrogen (TN) and ammonium nitrogen (NH_4_^+^-N) were analyzed by colorimetric methods (Hach Lange GmbH, Germany). The free ammonia (NH_3_-N) concentration was calculated as in Astals et al. [51]. Analyses were performed directly with the raw sample or with the soluble fraction by centrifuging the samples at 4600 rpm for 5 min and filtering the supernatant through a Rotilabo CME 0.45 μm nylon filter (Carl Roth GmbH, Germany). Specific volatile fatty acids (acetate, propionate, iso-butyrate, n-butyrate, iso-valerate and n-valerate) were analyzed using a Shimadzu GC-2010 plus Gas Chromatograph coupled to an FID detector and equipped with a Macherey-Nagel Optima FFA plus capillary column (Macherey-Nagel GmbH & Co. Germany).

### Bioreactor adaptation and start-up

The start-up of the bioreactor consisted of an adaptation period for the microbial community to the use of *Spirulina* as the main substrate. To do so, initially the reactor was fed with plain glucose (1.66 g L^−1^ day^−1^) which was gradually substituted for freeze dried *Spirulina*, following the substitution strategy from Vergara-Fernandez et al. [52] until only *Spirulina* was fed at 1.66 g L^−1^ day^−1^. Once the microbial community was adapted to *Spirulina*, the bioreactor was fed with 2.0 g *Spirulina* L^−1^ day^−1^ until the biogas production and the process parameters (alkalinity, TS and VS) were constant. In order to start the experiment with the lowest possible residual *Spirulina* biomass in the bioreactor’s sludge the feeding was stopped until the biogas production was below 100 mL day^−1^. After the starvation period, the study of the anaerobic digestion of freeze dried *Spirulina* began.

### Metagenome analysis

#### DNA sample preparation, sequencing and quality trimming

15.0 mL of sludge obtained from the bioreactor digesting freeze dried *Spirulina* were used for DNA extraction (sampling day 336, biogas production, 1676 mL biogas day^−1^, methane content 60 %). DNA was extracted according to Zhou et al*.* [53] with minor modifications. 2.5 μg of extracted DNA were used to prepare a 200 bp insert size sequencing library for the Ion Torrent Personal Genome Machine (PGM) platform (Life Technologies, USA). The instructions according to the Ion Xpress™ - Plus gDNA Fragment Library Preparation manual were followed, except for the initial DNA fragmentation, which was done using a GS FLX Standard Nebulizer Kit (Roche Applied Science, Germany), nebulization for 3 min at 32 psi. Sequencing template preparation was performed using the OneTouch Instrument and the OneTouch ES module. Enriched ISP particles were sequenced with the Ion PGM™ 200 Sequencing Kit (Life Technologies, USA) on a 318™ Chip with 520 flows following the manufacturer’s instructions. Automated analysis was performed with the Torrent Suite™ Software v3.2 using default settings. Additional quality filtering was done using the Trimmomatic tool v3 (http://www.usadellab.org/cms/index.php?page=trimmomatic) [54], with settings for removal of trailing bases of a q-value lower than 20, and removal of reads shorter than 50 bases.

#### Assembly of quality trimmed reads

Quality trimmed reads longer than 49 bp were assembled into contigs by means of the Genome Sequencer De Novo Assembler Software v2.6 (Newbler assembler, Roche Applied Science, Germany). In total, three read assemblies were performed, one with default settings for genomic DNA (assembly A), one with more stringent settings for better assembly of 16S rDNA sequences (assembly B), according to Fan et al., 2012 [55] and a third one (assembly C) with default settings but using only reads with a minimum length of 220 bp in order to better assemble Clostridial sequences. Additionally, EMIRGE [56] was used to reconstruct 16S rDNA fragments that did not assemble with our procedures.

#### In-depth taxonomy analysis

Contigs from assembly A were binned into provisional whole genome sequences of abundant populations in order to taxonomically analyze the microbial population. Contigs were binned, based on tetranucleotide pattern combined with interpolated Markov models, and submitted to a blast search [57] against a database containing all bacterial genomes downloaded from NCBI on May 2013 (ftp://ftp.ncbi.nlm.nih.gov/genomes/archive/old_genbank/Bacteria/) using the Metawatt v1.7 pipeline (http://sourceforge.net/projects/metawatt) (for further details concerning the binning pipeline see Strous et al*.* [18]). Binning options were set as follows: read length 200 nt; minimum bin size 100 kb and minimum contig size 500 bp. Generated bins were manually revised and assigned to a taxon by blasting all contigs from the selected bins against the 16S rRNA SILVA database [58]. Coverage and bin size of each particular bin were used to estimate the abundance of each population. Furthermore, transfer-RNAs of each bin were identified with ARAGORN [59] and the genome completeness for each population was estimated by the identification of 139 conserved Pfams as described by Campbell et al*.* [60].

#### Phylogeny of assembled 16S rDNA sequences

To identify 16S rDNA sequences among the assembled contigs, all contigs from the three assemblies were submitted to a blastn search against the RDP database (v10-32) [61]. Sequence parts with a hit were extracted and aligned parts with a minimum length of 1000 (Bacteria) or 500 bases (Archaea) were further analyzed. Together with the 16S rDNA fragments detected using EMIRGE, the assembled 16S rDNA sequences were submitted both to the RDP classifier [62] and the SINA classifier [63] with the confidence threshold or minimum sequence similarity set to 80 %, respectively. The sequences were also submitted to a blastn search against the current (Feb. 2014) NCBI nucleotide collection (nr/nt), and reference RNA sequences (refseq_rna). For both blastn searches the top blast hit for each query sequence was obtained. All sequences (contig parts, blast search hits, further representative 16S rDNA sequences) were aligned with muscle [64]. Phylogenetic trees were generated with FastTree [65] with the GTR + CAT model, bootstrapping (500 reps.) was done using seqboot (v3.67, http://evolution.genetics.washington.edu/phylip.html [66]), and the CompareToBootstrap.pl script (Price M. N., http://www.microbesonline.org/fasttree/treecmp.html) was used to implement the bootstrap values into the main tree. Trees were drawn using dendroscope [67].

#### Metagenome comparison

To determine if the substrate had any effect on the microbial community both in composition and function, a publicly available metagenome from a fully operational biogas plant treating mainly cellulose rich material: maize silage, 63 %; green rye, 35 % and low amounts of chicken manure, around 2 %, was downloaded from the NCBI database (SRR034130.1) [14] and compared to our lab scale metagenome.

To compare both metagenomes the same normalization procedure as in Jaenicke et al*.* [14] was applied to the 50 bp quality trimmed reads dataset which resulted in a dataset with 1,019,333 reads (*Spirulina*-S1). See Additional file 3: Material and Methods for further normalization details.

##### Taxonomic and functional comparison

*Spirulina*-S1 dataset and the biogas plant dataset, Maize-Rye dataset (M-R), were uploaded to the MG-Rast pipeline. Taxonomic analysis was done with the M5NR representative hit classification while functional analysis was done with the COG classification both with an e-value of 1e^−5^, 60 % minimum identity and 15 bp minimum length. The Mann–Whitney *U* test [68] was used to calculate the p value for the differences observed among the abundance of the classified COGs. Furthermore, specific protein domains (Pfam) related to cellulose degradation, and protein and amino acid degradation were identified in both datasets. In short, both datasets were first translated into amino acids and searched for open reading frames (ORFs) and subsequently blasted against the Pfam-A protein database [69]. See Additional file 3: Material and Methods for further details on the performance of the statistical analysis and the identification of ORFs.

##### Microbial diversity

To assess the microbial diversity of both populations, *Spirulina*-S1 and M-R, two approaches were used: (i) the determination of the evenness by Lorenz curves [46, 70] and (ii) the determination of the diversity by calculating Simpson’s diversity index (SDI) [47, 48]. These approaches were applied at species level (i) on the bacterial population and (ii) on the archaeal population.

### Accession numbers

Metagenomic reads and assembled contigs are accessible via NCBI under the Bioproject PRJNA239997. The sequenced reads were submitted to the Sequence Read Archive (http://www.ncbi.nlm.nih.gov/Traces/sra/) with the sample accession number SRS565943. Contigs of the tree assemblies (A, B and C) were submitted to GenBank, under the accession numbers JMBV00000000, JMBW00000000 and JMBX00000000. The versions described in this paper are versions JMBV01000000, JMBW01000000 and JMBX01000000. The sample numbers for the three assemblies are SAMN02727904, SAMN02727905, SAMN02727906; they are grouped in sample group SAMN02671764. The 11 16S rDNA sequences used for the generation of the phylogenetic trees in Fig. 2 were submitted to GenBank under the sample number SAMN03078811, with accession numbers KM851210-KM851220.

All metagenomes analyzed with the MG-Rast metagenome analyzer are publicly available with the following IDs: *Spirulina-S1* metagenome (4545162.3); Maize-Rye metagenome (4545349.3).

### Availability of supporting data

The datasets supporting the conclusions of this article are included within the article (and its additional files) while the phylogenetic data is available in the TreeBase repository http://purl.org/phylo/treebase/phylows/study/TB2:S18612.

## Additional files

Additional file 1: Figure S1. Contig assembly depth, GC content and taxonomic classification of selected bins. **Figure S2.** Phylogenetic tree of assembled Firmicutes 16S rDNA sequences from all three assemblies. **Figure S3.** Population evenness by means of Lorenz curves. (PDF 412 kb) Additional file 2: Table S1. RDP and SINA classification results for assembled and EMIRGE detected 16S rDNA sequences. **Table S2.** Assembly depth values and classification results of selected contigs encoding 16S rDNA sequences. **Table S3.** Taxonomic classification of metagenomic reads. **Table S4.** Identified Pfam related to cellulose degradation. **Table S5.** Identified Pfam related to protein degradation. **Table S6.** Identified Pfam related to amino acid degradation. **Table S7.** Accession numbers of reference 16S rDNA sequences in phylogenetic trees. (PDF 337 kb) Additional file 3:
**File contains additional information regarding the normalization of metagenomic datasets, the Mann-Whitney** ***U*** **test statistical test and the detection of specific protein domains.** (PDF 524 kb)

## Abbreviations

*Spirulina*-S1: Metagenomic sub-dataset 1 from the *Spirulina* reactor
M-R: Maize-Rye metagenome dataset
SDI: Simpson’s diversity index
S-CSTR: Semi-continuous stirred tank reactor
HRT: Hydraulic retention time
OLR: Organic loading rate
TS: Total solids
VS: Volatile solids
BOD_5_: Five day biological oxygen demand
COD_T_: Total chemical oxygen demand
COD_S_: Soluble chemical oxygen demand
TN: Total nitrogen
NH_3_-N: Ammonia nitrogen
NH_4_^+^-N: Ammonium nitrogen
VFAs: Volatile fatty acids
SBP: Specific biogas potential
SMP: Specific methane potential
