# Supplementary material for: Metagenome from a Spirulina digesting biogas reactor: analysis via binning of contigs and classification of short reads
Source: BMC Microbiol. 2015 Dec 17;15:277. doi: 10.1186/s12866-015-0615-1 (PMC4683858; doi:10.1186/s12866-015-0615-1)
Supplement: Additional file 1: Figure S1. — Contig assembly depth, GC content and taxonomic classification of selected bins. Figure S2. Phylogenetic tree of assembled Firmicutes 16S rDNA sequences from all three assemblies. Figure S3. Population evenness by means of Lorenz curves. (PDF 412 kb) [file 12866_2015_615_MOESM1_ESM.pdf]

Metagenome analysis and biogas production from the anaerobic digestion of the protein rich microalga *Spirulina*

V. Nolla-Ardèvol, M. Peces, M. Strous, H.E. Tegetmeyer

Supplementary Figures

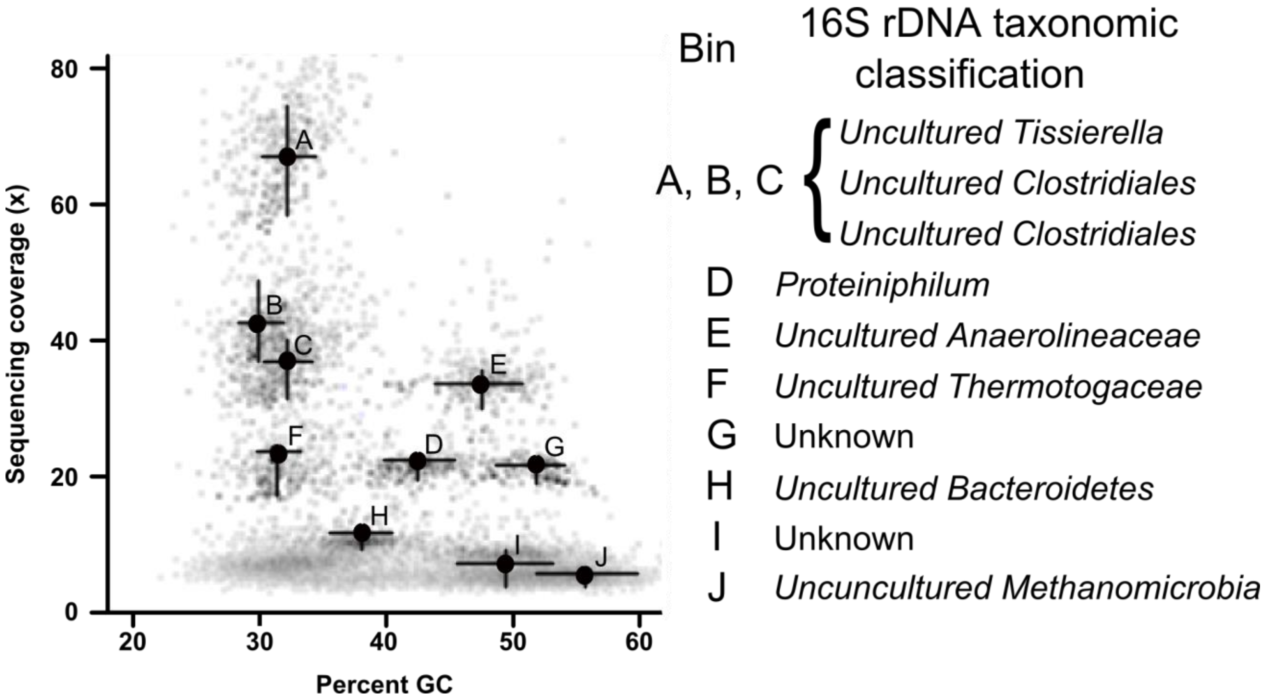

Suppl. Figure 1 Contig coverage, GC content and taxonomic classification of selected bins

Dot plot graph generated with the Metawatt v1.7 pipeline representing all the assembled contigs (grey) and the 10 selected bins (represented by black dots in the center of the bins, including bars showing contig distribution in each bin.)

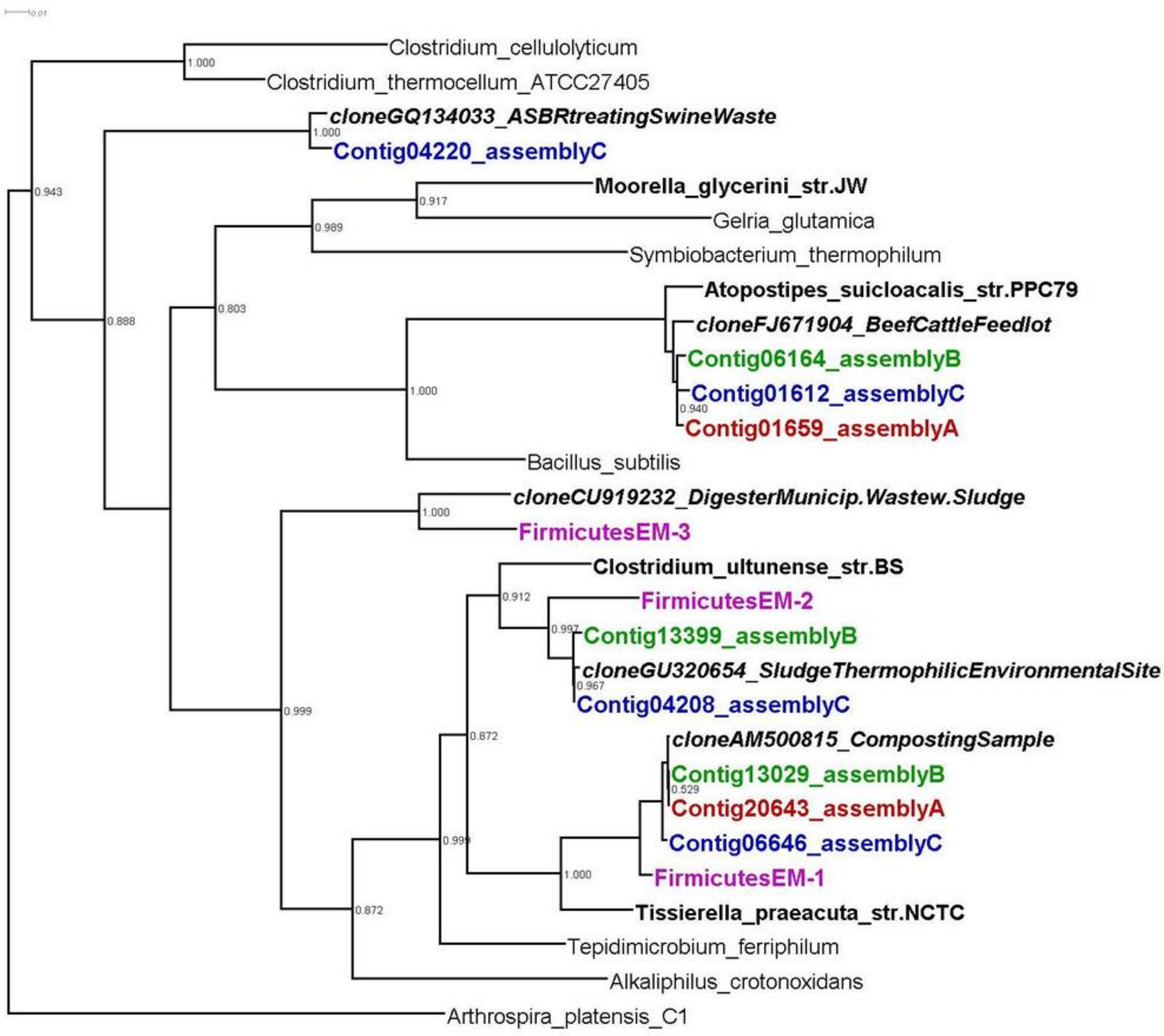

Suppl. Figure 2 16S rDNA Firmicutes phylogenetic tree

Includes the sequences of the Firmicutes branch in Figure 2a and additionally the unbinned contigs listed in Suppl. Table 2 that are not included in Figure 2a. Values at nodes are from the FastTree default Shimodaira-Hasegawa test.

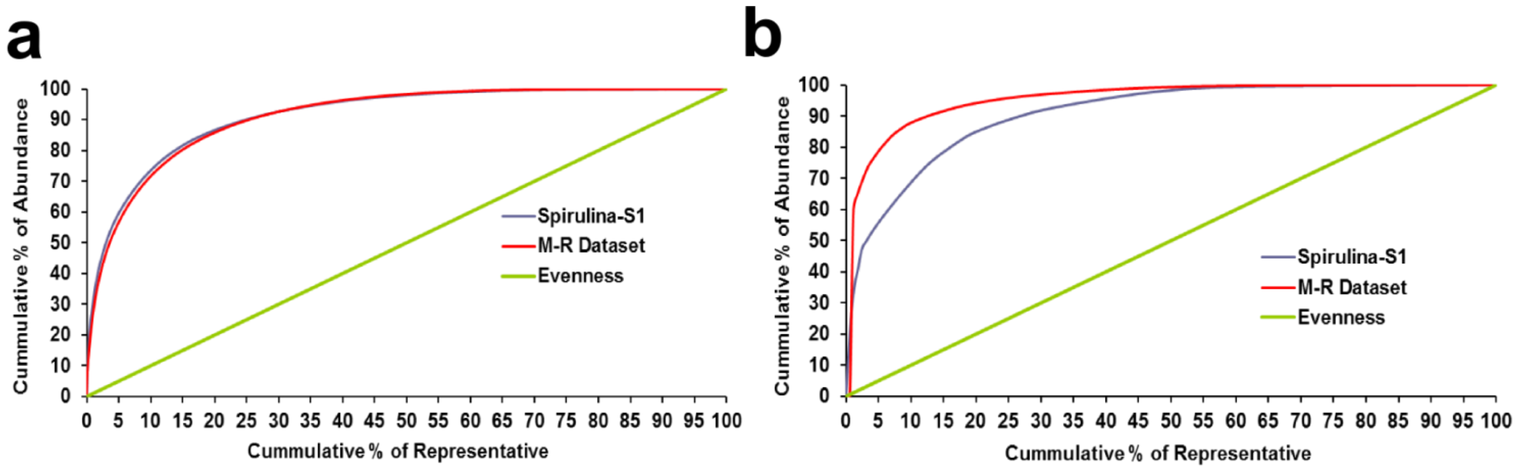

Suppl. Figure 3 Microbial population evenness

Lorenz curves for the estimation of population evenness at species level from the *Spirulina*-S1 and the Maize-Rye datasets. **a)** Bacterial evenness curves. **b)** Archaeal evenness curves.
