# Supplementary material for: Metagenome from a Spirulina digesting biogas reactor: analysis via binning of contigs and classification of short reads
Source: BMC Microbiol. 2015 Dec 17;15:277. doi: 10.1186/s12866-015-0615-1 (PMC4683858; doi:10.1186/s12866-015-0615-1)
Supplement: Additional file 2: Table S1. — RDP and SINA classification results for assembled and EMIRGE detected 16S rDNA sequences. Table S2. Assembly depth values and classification results of selected contigs encoding 16S rDNA sequences. Table S3. Taxonomic classification of metagenomic reads. Table S4. Identified Pfam related to cellulose degradation. Table S5. Identified Pfam related to protein degradation. Table S6. Identified Pfam related to amino acid degradation. Table S7. Accession numbers of reference 16S rDNA sequences in phylogenetic trees. (PDF 337 kb) [file 12866_2015_615_MOESM2_ESM.pdf]

Metagenome analysis and biogas production from the anaerobic digestion of the protein rich microalga *Spirulina*

V. Nolla-Ardèvol, M. Peces, M. Strous, H.E. Tegetmeyer

Supplementary tables

Suppl. Table 1 RDP and SINA classification results for assembled and EMIRGE detected 16S rDNA sequences

Classification of 16S rDNA sequences with RDP and SINA sequence classifiers. Sequences are assigned with a minimum of 80% confidence for the RDP, for SINA with a minimum of 80% identity to reference sequences and 10 LCA neighbors.

Sequence name	16S rDNA fragment length [bp]	Detected by/in	RDP classification (lowest classified rank)	SINA classification (lowest classified rank)
FirmicutesEM-1	1425	EMIRGE	Clostridiales: 1 / Clostridiales Incertae Sedis XI: 0.99 / *Tissierella* (genus): 0.99	Clostridiales; Family XI Incertae Sedis (family)
FirmicutesEM-2	1450	EMIRGE	Clostridiales: 1 / Incertae Sedis XI: 0.87 / Clostridium XII (genus): 0.87	Clostridiales; Family XI Incertae Sedis; *Tepidimicrobium* (genus)
FirmicutesEM-3	1357	EMIRGE	Clostridia: 1 / Clostridiales: 0.98 /Clostridiales_Incertae Sedis XI: 0.95 / *Tepidimicrobium* (genus): 0.85	Clostridia (class)
Contig01659	1559	Assembly A	*Atopostipes* (genus): 1	*Atopostipes* (genus)
Contig04220	1061	Assembly C	Bacteria (Domain): 1	Firmicutes; OPB54 (class)
Contig03630	1528	Assembly A	Thermotogaceae: 1 / *Petrotoga* (Genus): 1	Thermotogaceae (family)
Contig05284	1442	Assembly B	Anaerolineaceae (family): 1	Anaerolineaceae (family); uncultured
Contig00447	1512	Assembly C	Porphyromonadaceae: 1 / *Proteiniphilum* (genus): 0.99	Porphyromonadaceae (family)
Contig03141	1272	Assembly A	Bacteroidetes: 1 / Flavobacteria: 0.82 / Flavobacteriales: 0.82 / Cryomorphaceae (family): 0,81	Bacteroidetes; Sphingobacteriia; *Sphingobacteriales* (order)
Contig06470	925	Assembly A	Methanomicrobia: 1/ Methanosarcinales: 1 / *Methanosarcina* (genus): 1	Methanomicrobia (class)
Contig11126	551	Assembly B	*Methanoculleus* (genus): 1	*Methanoculleus* (genus)

Suppl. Table 2 Assembly depth values and classification results of selected contigs encoding 16S rDNA sequences

Contig classification according to RDP and SINA classifiers. Similarity to EMIRGE detected 16S rDNA sequences is based on alignment (see phylogenetic tree topology in Additional File-1 Suppl. Figure 2).

<table>
<tr><th>Bin</th><th>16S contig</th><th>Assembly</th><th>Cov*</th><th>RDP</th><th>SINA</th><th>EMIRGE</th><th>Bin Cov (X)</th></tr>
<tr><td rowspan="9">Unbinned</td><td>01659</td><td>A</td><td>11.4</td><td rowspan="3">Atopostipes</td><td rowspan="3">Atopostipes</td><td rowspan="3"></td><td rowspan="9">n.a</td></tr>
<tr><td>06164</td><td>B</td><td>8.4</td></tr>
<tr><td>01612</td><td>C</td><td>7.1</td></tr>
<tr><td>20643</td><td>A</td><td>158.9</td><td>Clostridiales, Inc. Sed. XI</td><td>Clostridiales, Inc. Sed. XI</td><td rowspan="3">EM-1</td></tr>
<tr><td>13029</td><td>B</td><td>76.5</td><td rowspan="2">Tissierella</td><td rowspan="2">Clostridiales, Inc. Sed. XI</td></tr>
<tr><td>06646</td><td>C</td><td>102.3</td></tr>
<tr><td>13399</td><td>B</td><td>141.4</td><td rowspan="2">Clostridiales, Inc. Sed. XI</td><td rowspan="2">Tepidimicrobium</td><td rowspan="2">EM-2</td></tr>
<tr><td>04208</td><td>C</td><td>164.2</td></tr>
<tr><td>04220</td><td>C</td><td>33.4</td><td>Clostridia</td><td>Firmicutes; OPB54</td><td></td></tr>
<tr><td rowspan="3">D</td><td>03740</td><td>A</td><td>38.2</td><td rowspan="3">Proteiniphilum</td><td rowspan="2">Proteiniphilum</td><td rowspan="2"></td><td rowspan="3">22.3</td></tr>
<tr><td>03861</td><td>B</td><td>25.8</td></tr>
<tr><td>00447</td><td>C</td><td>18.5</td><td>Porphyromonadaceae</td><td></td></tr>
<tr><td rowspan="2">E</td><td>05284</td><td>B</td><td>24.6</td><td rowspan="2">Anaerolineaceae</td><td rowspan="2">Unc. Anaerolineaceae</td><td rowspan="2"></td><td rowspan="2">33.3</td></tr>
<tr><td>02468</td><td>B</td><td>25</td></tr>
<tr><td rowspan="3">F</td><td>03630</td><td>A</td><td>50.3</td><td rowspan="3">Petrotoga</td><td rowspan="3">Thermotogaceae</td><td rowspan="3"></td><td rowspan="3">23.4</td></tr>
<tr><td>03105</td><td>B</td><td>34.4</td></tr>
<tr><td>03129</td><td>C</td><td>26.1</td></tr>
<tr><td rowspan="3">H</td><td>03141</td><td>A</td><td>8.9</td><td>Cryomorphaceae</td><td>Sphingobacteriales</td><td></td><td rowspan="3">11.5</td></tr>
<tr><td>10506</td><td>B</td><td>6.9</td><td>Flavobacteria</td><td>Bacteroidetes</td><td></td></tr>
<tr><td>02933</td><td>C</td><td>5.5</td><td>Cryomorphaceae</td><td>Sphingobacteriales</td><td></td></tr>
<tr><td colspan="8">* Assembly depth value from Newbler output
Unc: Uncultured</td></tr>
</table>

Suppl. Table 3 Taxonomic classification of metagenomic reads

Classification of reads from datasets "*Spirulina*-S1" and "MR" with the MG-Rast metagenome analyzer. Listed are the 10 most abundant bacteria and 3 most abundant archaea of both datasets based on the M5NR database with a $1e^{-5}$ E-value cutoff, a 60% minimum identity and 15 bp minimum length reads.

***Spirulina*-S1 dataset**				
**Class**	**Order**	**Family**	**Genus**	**Hits**
**Bacteria**				
Clostridia	Clostridiales	Clostridiaceae	*Alkaliphilus*	39255
			Clostridium	89870
Bacteroidia	Bacteroidales	Bacteroidaceae	*Bacteroides*	25932
Clostridia	Clostridiales	Peptostreptococcaceae	*Peptostreptococcaceae*	21781
Bacilli	Bacillales	Bacillaceae	*Bacillus*	20918
Thermotogae	Thermotogales	Thermotogaceae	*Petrotoga*	20262
Clostridia	Clostridiales	Fam XI Insertae Sedis	*Peptoniphilus*	12495
	Thermoanaerobacterales	Thermoanaerobacteraceae	*Caldanaerobacter*	11096
Anaerolineae	Anaerolineales	Anaerolineaceae	*Anaerolinea*	8033
Clostridia	Clostridiales	Peptococcaceae	*Desulfitobacterium*	6833
**Archaea**				
Methanomicrobia	Methanosarcinales	Methanosarcinaceae	*Methanosarcina*	5122
	Methanomicrobiales	Methanomicrobiaceae	*Methanoculleus*	3997
Methanococci	Methanococcales	Methanococcaceae	*Methanococcus*	431
**Maize-Rye dataset**				
**Bacteria**				
Clostridia	Clostridiales	Clostridiaceae	*Clostridium*	114140
Bacteroidia	Bacteroidales	Bacteroidaceae	*Bacteroides*	47051
Unclassified	Unclassified	Unclassified	*Candidatus Cloacamonas*	36032
Bacilli	Bacillales	Bacillaceae	*Bacillus*	32446
Clostridia	Clostridiales	Clostridiaceae	*Alkaliphilus*	18065
Bacteroidia	Bacteroidales	Porphyromonadaceae	*Parabacteroides*	16365
Clostridia	Clostridiales	Peptococcaceae	*Desulfotomaculum*	16053
			Desulfitobacterium	13461
			Pelotomaculum	12639
Bacilli	Bacillales	Bacillaceae	*Geobacillus*	11970
**Archaea**				
Methanomicrobia	Methanomicrobiales	Methanomicrobiaceae	*Methanoculleus*	54153
	Methanosarcinales	Methanosarcinaceae	*Methanosarcia*	4768
	Methanomicrobiales	Methanomicrobiaceae	*Methanoplanus*	4062

Suppl. Table 4 Identified Pfams related to cellulose degradation

Number of cellulose degradation Pfams identified with the hmmscan tool with a 1.0 E-value cutoff in each dataset.

Pfam	ID	Function	*Spirulina*-S1 dataset	M-R dataset*
PF00150	Cellulase	Hydrolyses glycosidic bond	61	304
PF00331	Glyco_hydro_10	Hydrolyses glycosidic bond	34	429
PF00404	Dockerin_1	Cellulosome structure	188	316
PF00457	Glyco_hydro_11	Hydrolyses glycosidic bond	2	36
PF00553	CBM_2	Carbohydrate binding module	15	80
PF00703	Glyco_hydro_2	Carbohydrate degradation	56	254
PF00704	Glyco_hydro_18	Hydrolyses glycosidic bond	150	309
PF00722	Glyco_hydro_16	Carbohydrate degradation	41	100
PF00734	CBM_1	Carbohydrate binding domain	78	139
PF00759	Glyco_hydro_9	Hydrolyses glycosidic bond	3	81
PF00840	Glyco_hydro_7	Hydrolyses glycosidic bond	3	11
PF00933	Glyco_hydro_3	Carbohydrate degradation	358	908
PF00942	CBM_3	Carbohydrate binding module	17	27
PF01183	Glyco_hydro_25	Carbohydrate degradation	31	37
PF01270	Glyco_hydro_8	Hydrolyses glycosidic bond	11	56
PF01341	Glyco_hydro_6	Hydrolyses glycosidic bond	1	16
PF01464	SLT	Carbohydrate degradation	169	320
PF02011	Glyco_hydro_48	Hydrolyses glycosidic bond	4	18
PF02013	CBM_10	Carbohydrate binding domain	24	34
PF02015	Glyco_hydro_45	Hydrolyses glycosidic bond	3	2
PF02018	CBM_4_9	Carbohydrate binding module	31	446
PF02055	Glyco_hydro_30	Hydrolyses glycosidic bond	14	67
PF02156	Glyco_hydro_26	Hydrolyses glycosidic bond	7	38
PF02836	Glyco_hydro_2_C	Hydrolyses glycosidic bond	202	483
PF02837	Glyco_hydro_2_N	Hydrolyses glycosidic bond	166	529
PF02839	CBM_5_12	Carbohydrate binding module	74	104
PF03422	CBM_6	Carbohydrate binding module	29	249
PF03442	CBM_X2	Carbohydrate binding module	5	79
PF03443	Glyco_hydro_61	Hydrolyses glycosidic bond	2	1
PF04616	Glyco_hydro_43	Hydrolyses glycosidic bond	72	1008
PF12891	Glyco_hydro_44	Hydrolyses glycosidic bond	4	21
PF14587	Glyco_hydr_30_2	Hydrolyses glycosidic bond	10	20
PF14600	CBM_5_12_2	Carbohydrate binding module	16	32

* M-R: Maize-Rye dataset

Suppl. Table 5 Identified Pfams related to protein degradation

Number of protein degradation Pfams identified with the hmmscan tool with a 1.0 E-value cutoff in each dataset.

Pfam	ID	Function	*Spirulina*-S1 dataset	M-R dataset*
PF00082	Peptidase_S8	Peptide degradation	770	900
PF00089	Trypsin	Protein degradation	198	425
PF00246	Peptidase_M14	Peptide degradation	117	207
PF00326	Peptidase_S9	Peptide degradation	496	1019
PF00413	Peptidase_M10	Peptide degradation	36	97
PF00450	Peptidase_S10	Peptide degradation	10	31
PF00557	Peptidase_M24	Peptide degradation	586	1054
PF01400	Astacin	Degradation of polypeptides	9	12
PF01431	Peptidase_M13	Peptide degradation	47	164
PF01434	Peptidase_M41	Peptide degradation	672	847
PF01447	Peptidase_M4	Peptide degradation	3	18
PF01546	Peptidase_M20	Peptide degradation	878	1276
PF01551	Peptidase_M23	Peptide degradation	801	1273
PF02031	Peptidase_M7	Peptide degradation	2	47
PF02868	Peptidase_M4_C	Peptide degradation	10	9
PF03070	TENA_THI-4	Secretion extracellular enzymes	10	31
PF03575	Peptidase_S51	Peptide degradation	37	78
PF04389	Peptidase_M28	Peptide degradation	219	531
PF05134	T2SL	Secretion of proteases	20	14
PF05257	CHAP	Peptidoglycan hydrolysis	31	60
PF05342	Peptidase_M26_N	Peptide degradation	12	32
PF05362	Lon_C	Peptide degradation	363	852
PF05543	Peptidase_C47	Peptide degradation	6	3
PF05548	Peptidase_M11	Peptide degradation	4	7
PF05569	Peptidase_M56	Peptide degradation	268	230
PF05576	Peptidase_S37	Peptide degradation	2	13
PF05577	Peptidase_S28	Peptide degradation	33	34
PF05649	Peptidase_M13_N	Peptide degradation	31	156
PF06480	FtsH_ext	Membrane bound protease	88	223
PF07502	MANEC	Protease activator and inhibitor	11	15
PF07580	Peptidase_M26_C	Peptide degradation	2	4
PF07687	M20_dimer	Peptide degradation	610	584
PF08548	Peptidase_M10_C	Peptide degradation	6	7
PF12388	Peptidase_M57	Peptide degradation	8	28
PF13365	Trypsin_2	Peptide degradation	288	539
PF13485	Peptidase_MA_2	Peptide degradation	245	370
PF13529	Peptidase_C39_2	Peptide degradation	109	129
PF13574	Reprolysin_2	Peptide degradation	40	43
PF13582	Reprolysin_3	Peptide degradation	51	93
PF13583	Reprolysin_4	Peptide degradation	19	43
* M-R: Maize-Rye dataset				

Suppl. Table 6 Identified Pfams related to amino acid degradation

Number of amino acid degradation Pfams identified with the hmmscan tool with a 1.0 E-value cutoff in each dataset.

Pfam	ID	Function	*Spirulina*-S1 dataset	M-R dataset*
PF00056	Ldh_1_N	Lactate/malate dehydrogenase	146	390
PF00070	Pyr_redox	Oxidoreductases	1254	2574
PF00155	Aminotran_1_2	Aminotransferase	1372	2613
PF00185	OTCace	Transferring one-carbon groups	282	482
PF00205	TPP_enzyme_M	Thiamine binding	134	460
PF00208	ELFV_dehydrog	Glutamate catabolism	590	882
PF00291	PALP	Amino acid metabolism	934	1024
PF01053	Cys_Met_Meta_P	Amino acid metabolism	1383	1166
PF01212	Beta_elim_lyase	Degradation of amino acids	767	1391
PF01571	GCV_T	Degradation of amino acids	334	375
PF02254	TrkA_N	Transporter	671	1608
PF02347	GDC-P	Degradation of amino acids	598	713
PF02729	OTCace_N	Transferring one-carbon groups	302	362
PF02812	ELFV_dehydrog_	Glutamate catabolism	304	289
PF02852	Pyr_redox_dim	Oxidoreductases	377	367
PF02866	Ldh_1_C	Lactate/malate dehydrogenase	37	111
PF03313	SDH_alpha	Degradation of amino acids	462	337
PF03315	SDH_beta	Degradation of amino acids	126	135
PF04898	Glu_syn_central	Amino acid metabolism	9	54
PF04960	Glutaminase	Amino acid metabolism	188	156
PF05995	CDO_I	Amino acid metabolism	16	22
PF07085	DRTGG	Amino acid metabolism	111	196
PF08669	GCV_T_C	Degradation of amino acids	90	183
PF12544	LAM_C	Amino acid metabolism	141	162
* M-R: Maize-Rye dataset				

Suppl. Table 7 Accession numbers of reference 16S rDNA sequences in phylogenetic trees

Accession numbers of reference 16S rDNA sequences for which in the phylogenetic trees (Fig. 2) only organism names are shown.

Organism name	Accession
Arthrospira platensis C1	gi|423061881:1247729-1249205
Tepidimicrobium ferriphilum strain SB91	gi|343198615|ref|NR_043077.1|
Atopostipes suicloacalis strain PPC79	gi|265678531|ref|NR_028835.1|
Clostridium ultunense strain BS	gi|219846939|ref|NR_026531.1|
Tissierella praeacuta strain NCTC 11158	gi|343206268|ref|NR_044860.1|
Gelria glutamica strain TGO	gi|343201113|ref|NR_041819.1|
Alkaliphilus crotonoxidans	gi|19072573|gb|AF467248.1|
Bacillus subtilis subsp. subtilis str. BSP1	gb|CP003695.1|:compl(3947493-3945959)
Symbiobacterium thermophilum IAM 14863	gi|444439729|ref|NR_075044.1|
Clostridium_cellulolyticum H10 strain H10	gi|507147961|ref|NR_102768.1|
Clostridium thermocellum ATCC 27405	gi|444304204|ref|NR_074629.1|
Moorella glycerini strain JW/AS-Y6	gi|265678890|ref|NR_029198.1|
Petrotoga mobilis SJ95 strain SJ95	gi|444303977|ref|NR_074401.1|
Levilinea saccharolytica strain KIBI-1	gi|343200285|ref|NR_040972.1|
Proteiniphilum acetatigenes strain TB107	gi|343202727|ref|NR_043154.1|
Owenweeksia hongkongensis DSM 17368	gi|470466026|ref|NR_074100.1|
Anaerolinea thermophila UNI-1 strain UNI-1	gi|444303960|ref|NR_074383.1|
Thermotoga_lettingae TMO strain TMO	gi|444439636|ref|NR_074951.1|
Bacteroides paurosaccharolyticus JCM 15092	gi|166063925|dbj|AB298727.2|
Alistipes finegoldii DSM 17242	gi|507148137|ref|NR_102944.1|
Fluviicola taffensis DSM 16823	gi|444304123|ref|NR_074547.1|
Methanosarcina siciliae strain T4/M	gi|559795168|ref|NR_104757.1|
Methanoculleus marisnigri JR1 strain JR1	gi|470467424|ref|NR_074174.1|
Methanospirillum hungatei JF-1 strain JF-1	gi|470467480|ref|NR_074177.1|
Methanobacterium flexile strain GH	gi|304336835|gb|EU333914.2|
Methanosaeta concilii strain Opfikon	gi|254971324|ref|NR_028242.1|
Methanocorpusculum parvum strain DSM 3823	gi|343206139|ref|NR_044728.1|
Methanothermobacter marburgensis strain Marburg	gi|254971323|ref|NR_028241.1|
Methanolobus oregonensis strain WAL1	gi|254971319|ref|NR_028237.1|
Methanococcus maripaludis	gi|1145365|gb|U38484.1|
Methanopyrus kandleri AV19 strain AV19	gi|444304115|ref|NR_074539.1|
