# Supplementary material for: Metagenome from a Spirulina digesting biogas reactor: analysis via binning of contigs and classification of short reads
Source: BMC Microbiol. 2015 Dec 17;15:277. doi: 10.1186/s12866-015-0615-1 (PMC4683858; doi:10.1186/s12866-015-0615-1)

# Metagenome analysis and biogas production from the anaerobic digestion of the protein rich microalga *Spirulina*

V. Nolla-Ardèvol, M. Peces, M. Strous, H.E. Tegetmeyer

## Supplementary Material and Methods

### 1. Metagenome normalization and comparison

In order to compare our metagenomic data with a publicly available metagenome, (SRR034130.1) (Jaenicke et al., 2011), normalization had to be applied to our data. Normalization of Dataset-1 was performed as in Jaenicke *et al.,* (Jaenicke et al., 2011). During the normalization procedure, reads shorter than 100 bp and longer than 309 bp were removed. From the initial 5,240,830 reads, after normalization, 2,486,976 reads remained. Subsequently from these 2.4 million reads, the same amount of reads as in the Jaenicke *et al.,* dataset, 1,019,333, were randomly selected in triplicates, generating data sets *Spirulina*-S1, S2 and S3. To make sure that the three randomly generated datasets were not biased, they were imported and analyzed using the MGX platform, a metagenomics platform currently being developed at the CeBiTec, Bielefeld University. The MGX platform employs the Conveyor workflow engine (Linke et al., 2011) for executing all analysis tasks.

As can be seen in Suppl. Material and Methods Figures 1a, b, c and d, no differences between the three randomly generated datasets were detected for any of the analyzed parameters. *Spirulina*-S1 dataset was chosen for the comparison with the public available metagenome. The two compared datasets,

*Spirulina*-S1 and M-R were also analyzed with the MGX platform in terms of read length and GC content (Suppl. Material and Methods Fig. 1e, f).

**2. Mann-Whitney U test**

A statistical analysis was used to determine if the observed differences between the two data sets, *Spirulina*-S1 and M-R for the numbers of reads assigned to a particular category of COGs were significant. First, for each COG the number of assigned reads was transformed to a percentage value of the total number of reads that were assigned to all COGs for each of the two data sets. The next step was to determine if these percentage values were normally distributed using the Shapiro-Wilk Normality Test in R. As the results showed that the distribution of the values was non-normal (both for all COGs and the subsets of COGs in the selected categories), the Mann-Whitney U tests (M-W) (Mann and Whitney, 1947) was applied to test for differences between the data set for the different COG categories. To perform the test, first all COGs that represented less than 0.001 % of abundance were removed from the datasets. Subsequently the M-W test was done for each selected category of COGs, first as a two sided test to determine if the percentage value distributions of the two datasets were different from each other and secondly as a one-side test to determine, if there were greater or smaller values in the *Spirulina*-S1 dataset compared to the Maize-Rye dataset.

**3. Generation of ORF and identification of specific protein domain (Pfam)**

Specific protein domains (Pfams) were searched in the randomly generated dataset *Spirulina*-S1 and the biogas plant dataset, Maize-Rye dataset (M-R). To do so, the following procedure was applied. First, all reads were translated to

amino acids and searched for ORFs with the "Translate DNA" script (Translatedna v 1.75 www.mbari.org/staff/haddock/scripts/) with the "0" option, "print all possible ORF for each read". Second, to identify Pfams among the reads, the resulting ORFs were blasted against the Pfam-A database (Punta et al., 2012) with the hmmscan-v3 tool (http://hmmer.org/) with the –E and - -domE values set to 1.0. Third, Pfams identifiers of particular interest were obtained from two sources; (i) directly from the Pfam database (http://pfam.sanger.ac.uk/) with the search terms "extra cellular proteases", "cellulases" and "cellulosome" and (ii) Pfams from genes involved in the Protein, Amino acid and Cellulose degradation pathways from the MetaCyc Database of metabolic pathways (Caspi et al., 2012). Subsequently the list of desired Pfams was searched amongst the Pfam domains identified in the hmmscan search.

## References


Caspi R, Altman T, Dreher K, Fulcher C a, Subhraveti P, Keseler IM, Kothari A, Krummenacker M, Latendresse M, Mueller L a, Ong Q, Paley S, Pujar A, Shearer AG, Travers M, Weerasinghe D, Zhang P, Karp PD. 2012. The MetaCyc database of metabolic pathways and enzymes and the BioCyc collection of pathway/genome databases. *Nucleic Acids Res.* **40**:D742–53.

Jaenicke S, Ander C, Bekel T, Bisdorf R, Dröge M, Gartemann K-H, Jünemann S, Kaiser O, Krause L, Tille F, Zakrzewski M, Pühler A, Schlüter A, Goesmann A. 2011. Comparative and joint analysis of two metagenomic datasets from a biogas fermenter obtained by 454-pyrosequencing. *PLoS One* **6**:e14519.

Linke B, Giegerich R, Goesmann A. 2011. Conveyor: a workflow engine for bioinformatic analyses. *Bioinformatics* **27**:903–11. http://www.ncbi.nlm.nih.gov/pubmed/21278189.

Mann HB, Whitney DR. 1947. On a test of whether one of two random variables is stochastically larger than the other. *An. Math. Stat.* **18**:50–60.

Punta M, Coggill PC, Eberhardt RY, Mistry J, Tate J, Boursnell C, Pang N, Forslund K, Ceric G, Clements J, Heger A, Holm L, Sonnhammer ELL, Eddy SR, Bateman A, Finn RD. 2012. The Pfam protein families database. *Nucleic Acids Res.* **40**:D290–301.

**Supplementary Material and Methods Figure 1 Datasets comparison with the MGX platform**

Comparison of the three randomly generated *Spirulina* datasets in terms of **(a)** Read length; **(b)** GC %; **(c)** 25 most abundant Phyla; **(d)** 20 most abundant COGs; and comparison of the *Spirulina*-S1 and the Maize-Rye dataset in **(e)** terms of read length and **(f)** GC %.

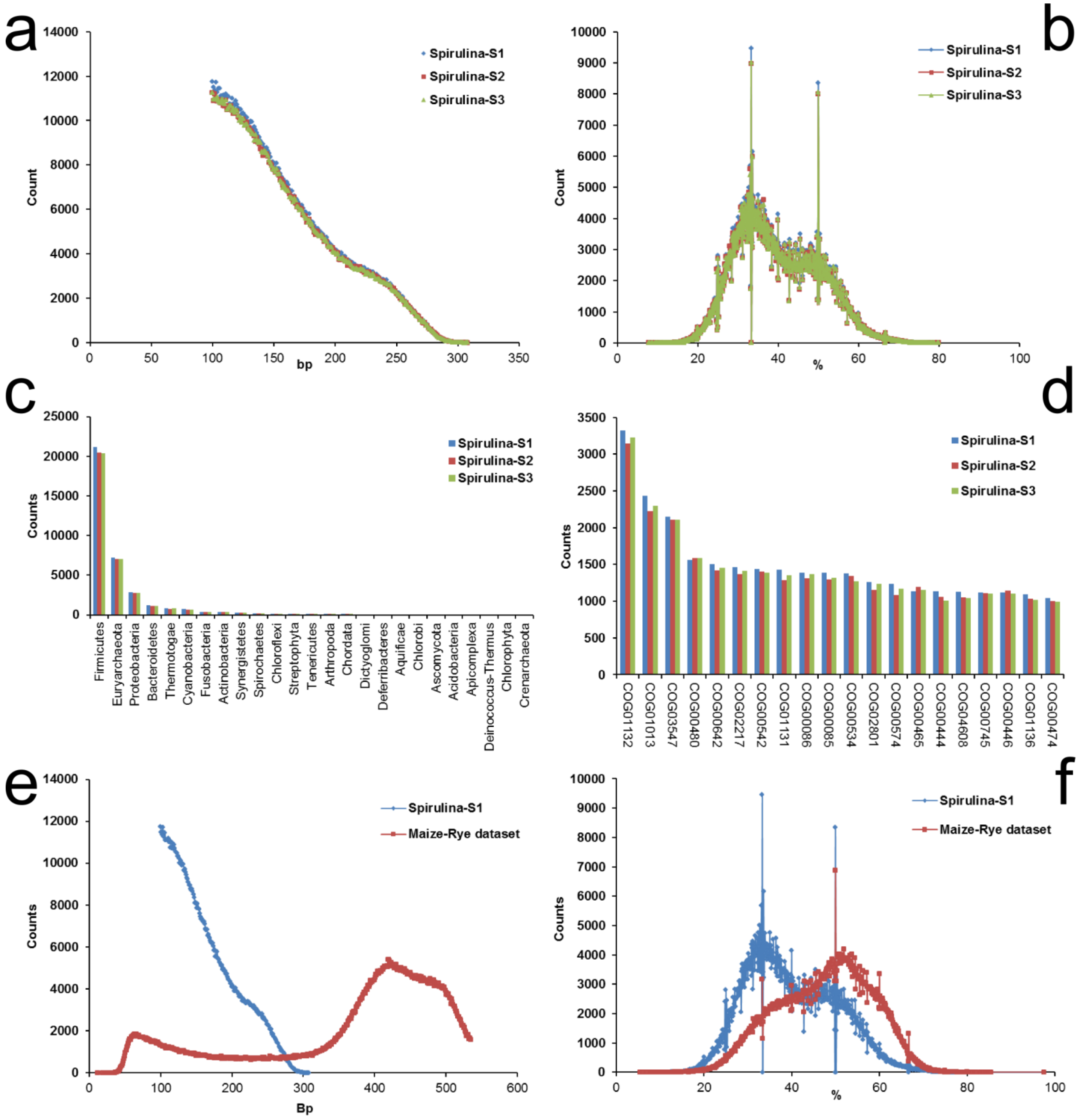

Supplement: Additional file 3: — File contains additional information regarding the normalization of metagenomic datasets, the Mann-Whitney U test statistical test and the detection of specific protein domains. (PDF 524 kb) [file 12866_2015_615_MOESM3_ESM.pdf]
